# Generation of novel in vitro flexible kidney organoid model to investigate the role of extracellular vesicles in induction of nephrogenesis

**DOI:** 10.1186/s12964-023-01374-z

**Published:** 2023-12-18

**Authors:** Naveed Ahmad, Anatoliy Samoylenko, Ichrak Abene, Eslam Abdelrady, Artem Zhyvolozhnyi, Olha Makieieva, Geneviève Bart, Ilya Skovorodkin, Seppo J Vainio

**Affiliations:** 1 Laboratory of Developmental Biology, Faculty of Biochemistry and Molecular Medicine, University of Oulu, 90220 Oulu, Finland; 2 Infotech Oulu, University of Oulu, 90014 Oulu, Finland; 3 Flagship GeneCellNano, University of Oulu, 90220 Oulu, Finland; 4 Kvantum Institute, University of Oulu, 90014 Oulu, Finland

**Keywords:** Nephrogenesis, UB, MM, Wnt, EVs, UB cell line, RalA, RalB

## Abstract

**Background:**

During kidney organogenesis, metanephric mesenchyme (MM) and ureteric bud (UB) interact reciprocally to form nephrons. Signaling stimuli involved in these interactions include Wnts, growth factors and nano/micro particles. How UB and MM are interacting is not completely understood. Our study investigated the signaling and communication via extracellular vesicles (EVs) during nephrogenesis. Embryonic day (E) 11.5 mouse kidney UB and MM produce very low number of primary cells that have limited ability for proliferation in culture. Such limitations obstruct studying the role of EVs in induction of nephrogenesis. These issues necessitate to generate a nephrogenesis model allowing to study the comprehensive role of EVs during nephrogenesis.

**Results:**

Our study generated a UB derived cell line-based in vitro flexible model of nephrogenesis allowing expandable cell culturing, in addition to performing characterization, tracking and blocking of EVs. UB cell line aggregation with E11.5 MM cells induced the formation of segmented nephrons. Most efficient nephrogenesis was obtained by the co-culturing of 30,000 cells of UB cell line with 50,000 MM cells. Results revealed that both the UB and the MM secrete EVs during nephrogenesis. UB cell line derived EVs were characterized by their size, morphology and expression of markers (CD63, TSG101, CD9 and CD81). Furthermore, proteomics data of UB cell line-derived EVs revealed large number of proteins involved in nephrogenesis-related signaling pathways. Palmitoylated GFP-tagged EVs from UB cell line were found in the nephron formation zone in the developing kidney organoid. UB cell line derived EVs did not induce nephrogenesis in MM cells but significantly contributed to the survival and nephrogenesis-competency of MM cells. The secretion of EVs was continuously inhibited during the ongoing nephrogenesis by the knockdown of RalA and RalB gene expression using short hairpin RNAs. This inhibition partially impaired the ability of UB cell line to induce nephrogenesis. Moreover, impaired nephrogenesis was partially rescued by the addition of EVs.

**Conclusion:**

Our study established a novel in vitro flexible model of nephrogenesis that solved the limitations of primary embryonic kidney cells and mouse embryonic stem cell kidney organoids for the EV research. EVs were found to be an integral part of nephrogenesis process.

**Graphical Abstract:**

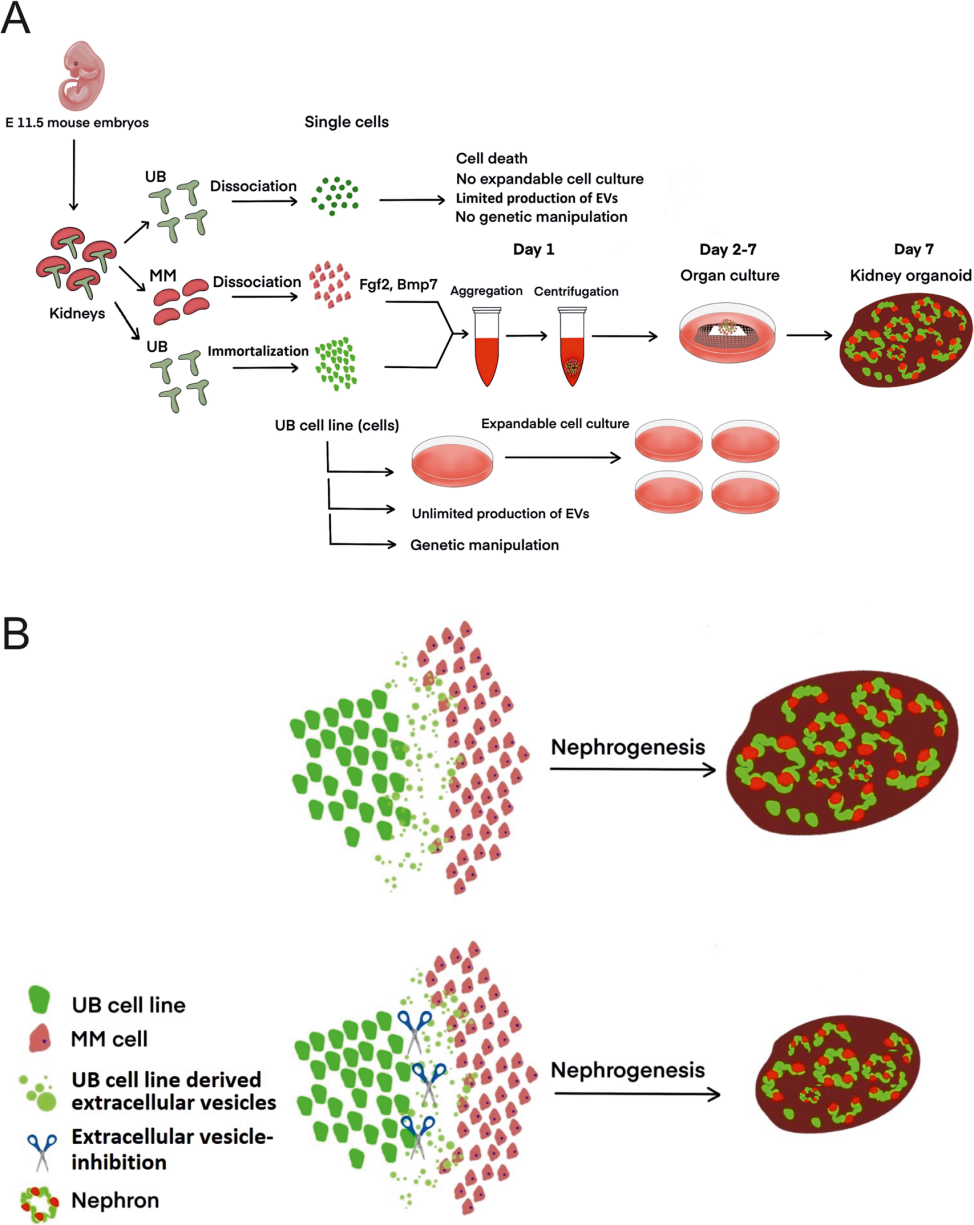

Video Abstract

**Supplementary Information:**

The online version contains supplementary material available at 10.1186/s12964-023-01374-z.

## Introduction

Cells secrete different membranous vesicles, which are collectively termed as extracellular vesicles (EVs). Exosomes and microvesicles (MVs) are the main types of EVs and recently a new type of EVs was discovered called exomere [1]. Exosomes are formed in multivesicular bodies (MVBs) and they are secreted by exocytosis when MVBs are fused with the plasma membrane [1]. MVs are formed by the outward budding of plasma membrane. Both exosomes and MVs carry deoxyribonucleic acid (DNA), ribonucleic acid (RNA), proteins and lipids [1, 2]. EVs were found crucial for cell–cell communication as EVs transport and exchange proteins, nucleic acids and lipids to induce different responses in the target cells and tissues [1].

With the limited available data, EVs were proposed to have a role in embryonic development [3–5]. EVs were shown to carry organ development signaling molecules, although the function of EVs remained obscure in organ development [6–9]. Considering the involvement of EVs in embryonic development, our study investigates the role of EVs in nephrogenesis. Nephron formation (nephrogenesis) is happening due to the interaction of ureteric bud (UB) and metanephric mesenchyme (MM). This interaction is reciprocal between the UB and MM, and many signaling molecules are exchanged during nephrogenesis in mouse embryonic kidney [10]. The signaling molecules include Wnt proteins, cell growth factors and other inducer molecules [11]. The role of EVs can be important compared to the Wnts and the growth factors (GF) including Bmp7, Fgf2 and GDNF, which were previously known in kidney development [11]. How the UB and MM cells interact is not completely understood.

The current study investigated how the UB and the MM interact via EVs, and how the EV-based interaction contribute to nephrogenesis. The primary mouse UB and MM cells have limitations with the low cell numbers available for cell culturing [12]. In addition, the primary tissues/cells cannot be effectively utilized for EV-targeted genetic modification, isolation of EVs in large quantity, tracking of EVs and genetic blocking of EV secretion to investigate the complex role of EVs in nephrogenesis. Mouse embryonic stem cell models can be used as a replacement of in vivo and ex vivo methods of nephrogenesis, but those models are not easily applicable to EV research due to being complicated, expensive and not suited for long-term cell cultures [13, 14]. We developed an efficient and easy way to model nephrogenesis, which allowed efficient genetic modification, detailed characterization of EVs, isolation of EVs in large quantity for the treatment of nephron forming MM cells, tracking of EVs and genetic blocking of EVs during nephrogenesis. This model is referred as in vitro flexible kidney organoid model of nephrogenesis, and our study aimed to investigate the role of EVs in nephrogenesis using this model.

## Results

### EV secretion by the kidney MM and UB cells

**Fig. 1:**
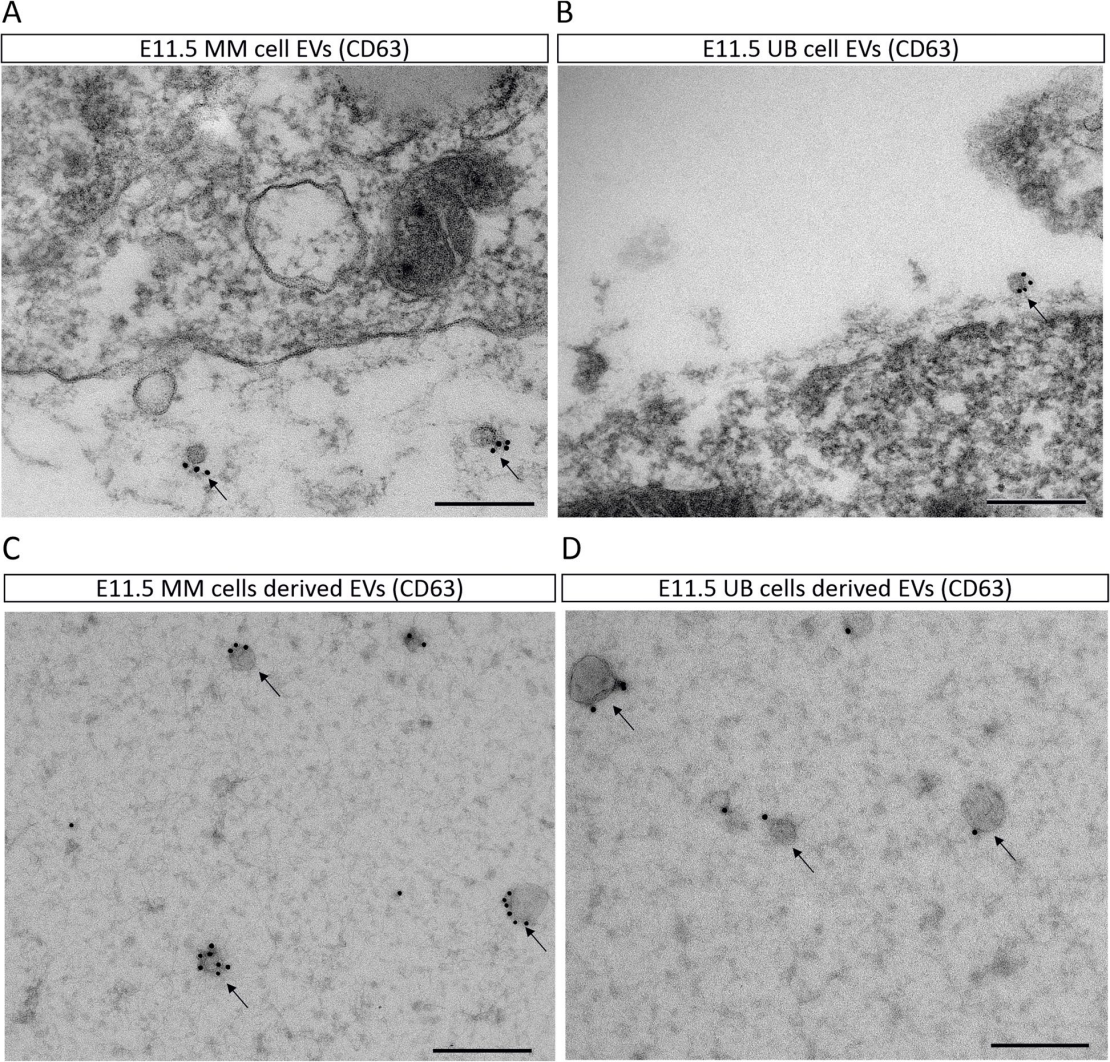
EV secretion by the mouse embryonic (E11.5) kidney MM and UB cells. **A** EVs (pointed with arrow) in the vicinity of E11.5 MM cell, immunolabelled with anti CD63 antibody. **B** EVs (pointed with arrow) in the vicinity of E11.5 UB cell, immunolabelled with anti CD63 antibody. **C**, **D** Immunolabelling of EVs (pointed with arrow) with anti CD63 antibody, secreted by monolayer-cultured MM (**C**) and UB cells (**D**). Scale bars: 200 nm

During nephrogenesis, EV secretion was investigated by the immunolabelling of EVs with anti CD63 antibody in E11.5 kidneys. Results showed the presence of EVs in the vicinity of both MM and UB cells as shown in immunoelectron microscopy of kidney sections (Fig. 1A-B). The secretion of EVs was further investigated in the cultured E11.5 kidney cells. Dissociated MM cells were provided with Bmp7 and Fgf2, whereas UB cells were provided with GDNF in separate culturing. EVs were isolated from both cultures. The study showed that both MM and UB cells secreted EVs (Fig. 1C-D). Isolated EVs were labelled with anti CD63 antibody, and their size was estimated to be 30—120 nm. EVs were shown to have circular shape (Fig. 1A-D). Our study showed that EVs are secreted by both MM and UB cells in nephrogenesis.

E11.5 MM and UB tissues formed nephrons when they were recombined after separation. The separation and recombination of MM and UB allowed limited cell culturing and functional genetic targeting of kidney development [12]. In our study, we extended the culturing time period to maximum 48 h by providing GF (Bmp7 and Fgf2) to MM and UB cells, to culture them and to subsequently isolate the EVs. However, the utilization of this separation and recombination model is not applicable to EV research and efficient genetic manipulation. Investigating the role of EVs in nephrogenesis using this model is problematic due to the very low number of primary cells obtained from E11.5 kidneys. In addition, due to short time survival and culturing problems of E11.5 UB and MM tissues/cells, in our study, we worked toward generating a flexible model of nephrogenesis, which can be utilized to investigate the complex role of EVs in nephrogenesis. The flexible model of nephrogenesis is aimed to resolve the limitations of E11.5 UB and MM cultures.

### Generation of in vitro flexible nephrogenesis model

**Fig. 2:**
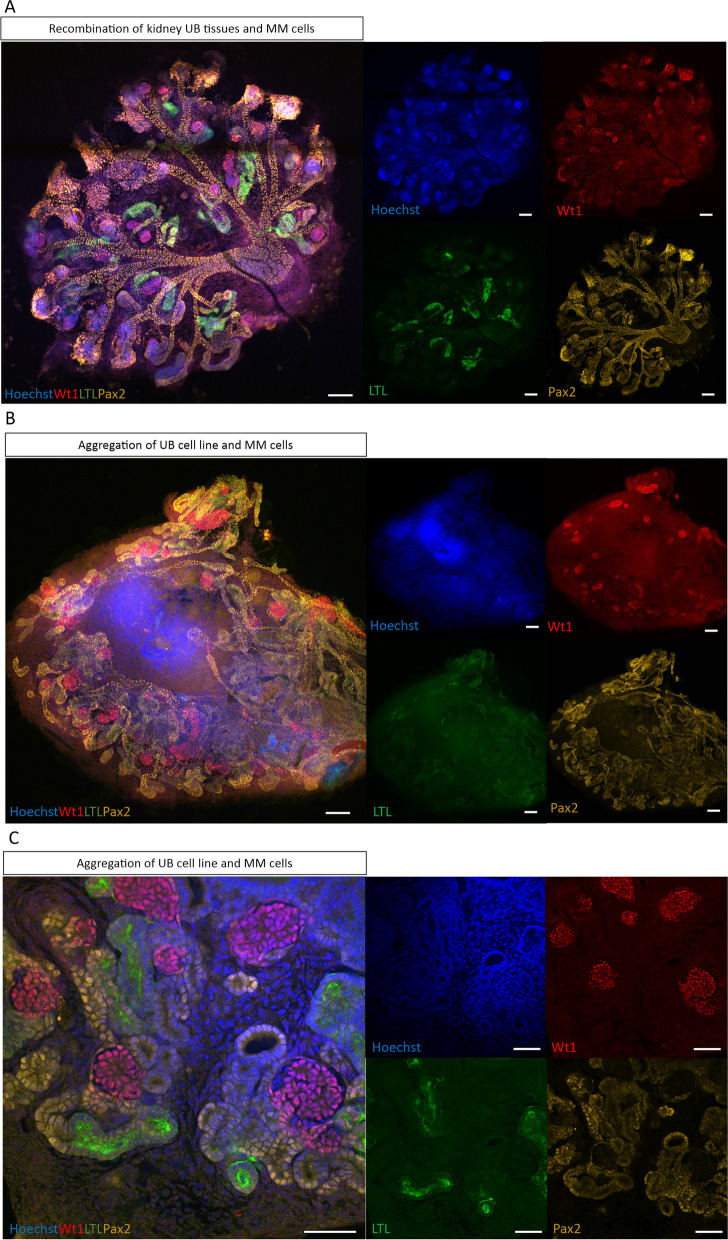
Generation of in vitro flexible model of nephrogenesis by the aggregation of the UB cell line and E11.5 MM cells. **A** Immunofluorescence confocal imaging of the control organoid showing nephrogenesis by staining with markers of segmented nephrons: Wt1—glomeruli, LTL—proximal tubules, Pax2—proximal and distal tubules, and Hoechst—nuclei. Organoid was generated by recombining five E11.5 UB tissues and 100,000 MM cells, and subsequently culturing organoid for a week. Control organoid is shown with the merged and separated channels (Hoechst-blue, Wt1-red, LTL-green and Pax2-yellow). Scale bars: 100 μm. **B** Immunofluorescence of the organoid generated by the co-culturing of UB cell line (50,000 cells) and MM cells (100,000) for one week. Segmented nephrons were shown with the markers of nephrogenesis: Wt1—glomeruli, LTL—proximal tubules, Pax2—proximal and distal tubules, and Hoechst – nuclei. Nephrons in the organoid are shown with the merged and individual channels (Hoechst-blue, Wt1-red, LTL-green and Pax2-yellow). Scale bars: 100 μm. **C** Confocal images of Immunofluorescence of segmented nephrons in organoid generated by the aggregation of the UB cell line and the E11.5 MM cells showing nephron markers with merged and separated channels (Hoechst-blue, Wt1-red, LTL-green and Pax2-yellow) in the figure. Scale bars: 50 μm

Considering the constraints of mouse primary kidney models/cells, our study aimed to generate an in vitro flexible model of nephrogenesis. We used UB cell line gifted by Barasch´s lab, Columbia University, United States, which were co-cultured with the dissociated E11.5 MM cells in the presence of Bmp7 and Fgf2. In the experiment, as a control, five E11.5 UB tissues were aggregated with 100,000 MM cells in the presence of Bmp7 and Fgf2. Organoids were cultured for one week in 3D Trowel organ culture system. Results showed full-blown nephrogenesis confirmed by staining with segmented nephron markers (Wt1—glomerulus, LTL—proximal tubule and Pax2—proximal and distal tubule) (Fig. 2A). Interestingly, the aggregation of UB cell line (50,000 cells) with E11.5 MM cells (100,000 cells) showed successful nephrogenesis, confirmed by staining with markers of nephrogenesis (Wt1—glomerulus, LTL—proximal tubule and Pax2—proximal and distal tubule) (Fig. 2B). Furthermore, our results confirmed that the nephrons generated by UB cell line are structured in correct geometrical shape. At higher magnification, confocal imaging showed correctly organized segmentation of glomerulus (Wt1-red), proximal tubule (LTL-green and Pax2-yellow) and distal tubule (Pax2-yellow) (Fig. 2C). Therefore, we confirmed that UB cell line is capable to stimulate the formation of segmented nephrons in MM cells.

### Optimization of cell number for the induction of efficient nephrogenesis

**Fig. 3:**
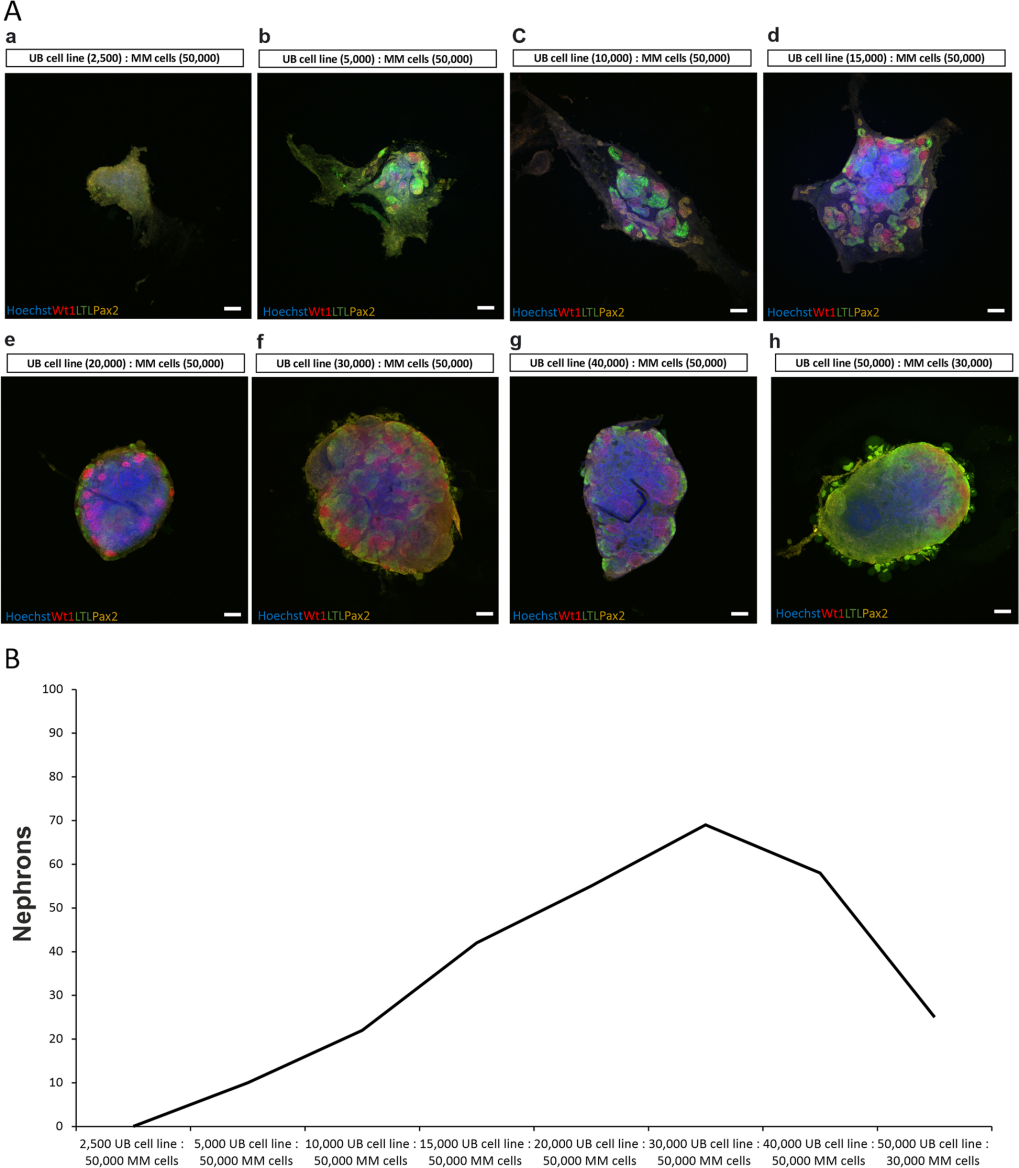
Different ratios of UB cell line and MM cells for nephrogenesis. **A** Confocal immunofluorescence images showing different ratios of combining UB cell line and MM cells for the induction of efficient nephrogenesis: (**a**) 2,500 UB cell line and 50,000 MM cells, (**b**) 5,000 UB cell line and 50,000 MM cells, (**c**) 10,000 UB cell line and 50,000 MM cells, (**d**) 15,000 UB cell line and 50,000 MM cells, (**e**) 20,000 UB cell line and 50,000 MM cells, (**f**) 30,000 UB cell line and 50,000 MM cells, (**g**) 40,000 UB cell line and 50,000 MM cells and (**h**) 50,000 UB cell line and 30,000 MM cells. Nephrogenesis was confirmed by staining organoids with nephron markers: Wt1 (glomeruli)—red, LTL (proximal tubules) – green and Pax2 (proximal and distal tubules)—yellow. Scale bars: 100 μm. **B** Graph showing the number of nephrons in organoids generated with different ratios of UB cell line and MM cells

We extended this study to thoroughly characterize our in vitro model of nephrogenesis. We tested different ratios of UB cell line and MM cells for the generation of organoids. Nephrogenesis in organoids was confirmed by staining organoids with nephron markers: Wt1—glomerulus, LTL—proximal tubule, Pax2—proximal and distal tubule (Fig. 3Aa-h). Results revealed that 2,500 UB cell line cells were not able to induce the formation of nephrons in 50,000 MM cells (Fig. 3Aa, Fig. 3B), although several nephrons (approximately 10) were induced in MM cells when the number of UB cell line cells was increased to 5,000 (Fig. 3Ab, B). The number of nephrons in organoids was gradually increased to approximately 22, 42 and 55 by increasing the number of UB cell line cells to 10,000, 15,000 and 20,000, respectively (Fig. 3Ac-e, B). The maximum number of nephrons (approximately 69) were formed when 30,000 UB cell line cells were mixed with 50,000 MM cells, but the number of nephrons started decreasing (approximately 58) when the number of UB cell line cells was further increased to 40,000 in the same number of MM cells (Fig. 3Af-g, B). The nephrogenesis was dramatically impaired and the number of nephrons was decreased to approximately 25 when the number of UB cell line cells was further increased to 50,000 in 30,000 MM cells (Fig. 3Ah, B). This experiment suggested that efficient nephrogenesis is attained when the organoid is generated by the aggregation of 30,000 UB cell line cells and 50,000 MM cells.

### Characterization of UB cell line derived EVs

**Fig. 4:**
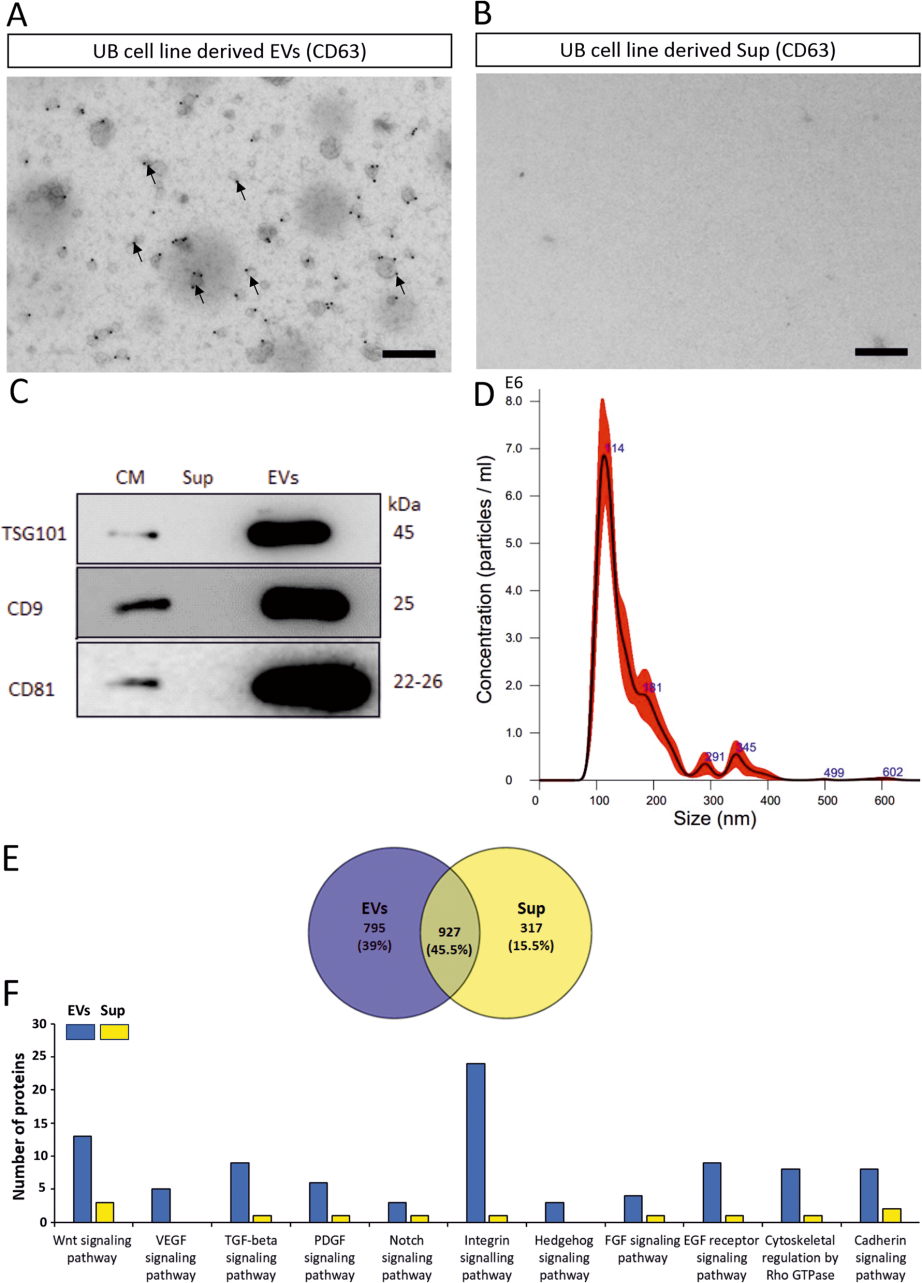
Comprehensive characterization of UB cell line derived EVs. **A** Electron microscopy showing UB cell line derived EVs immunolabelled with anti CD63 antibody. Several EVs labelled with anti CD63 antibody are pointed with arrows. Scale bar: 200 nm. **B** UB cell line derived Sup is shown in immunoelectron microscopy image. Staining of Sup sample was done with anti CD63 antibody. Scale bar: 200 nm. **C** Western blot with anti TSG101 (45 kDa), CD9 (25 kDa) and CD81 (22–26 kDa) antibodies against the standard EV markers in conditioned medium (CM), Sup and EVs. Original images for the Western blot are shown in the supplementary figures. **D** Concentration and distribution of UB cell line derived EVs are shown by NTA. **E** Venn diagram representing the shared and unique proteins between EVs and Sup. **F** Bar chart showing the number of proteins in EV and Sup involved in different nephrogenesis related signaling pathways. The lists of proteins in EVs and Sup involved in different nephrogenesis related signaling pathways are presented in supplementary tables 4 and 5

EVs from UB cell line were inspected using electron microscopy by immunolabelling with anti CD63 antibody. The data showed that the size of UB cell line EVs ranged from 30 to 150 nm and EVs were found to have round morphology as pointed with arrows. The majority of UB cell line derived EVs expressed the typical EV marker CD63 (Fig. 4A). UB cell line derived supernatant (Sup) had no EVs or expression of CD63, as confirmed by immunoelectron microscopy (Fig. 4B).

The EVs were further characterized by Western blot using antibodies against the EV markers (TSG101, CD9 and CD81). Results revealed the expression of TSG101, CD9 and CD81 EV markers in UB cell line derived EVs. Our results showed no expression of EV markers in UB cell line derived supernatant (Sup) sample, confirming the absence of EVs in Sup samples. Moreover, UB cell line derived conditioned medium (CM) showed the expression of standard EV markers (TSG101, CD9 and CD81), although less than that of EVs, hence the proportion of EVs in CM is less than that of EV samples (Fig. 4C).

The size and the number of UB cell line derived EVs were measured by NTA. The EVs had the mean size 159.5 ± 6.6 nm and showed distinct size peak at 114 nm. Our NTA data determined that the UB cell line derived EV sample contain 4.70 × 10^8^ ± 2.45 × 10^7^ particles/ ml (Fig. 4D). Size distributions shown by the immunoelectron microscopy analysis were in line with NTA data, except for the smaller sized EVs not detected by NTA.

Mass spectrometry-based proteomics of UB cell line EVs and Sup was performed, and proteomics data was analyzed by PANTHER bioinformatics tool. Venn diagram of proteomics study showed that UB cell derived EVs had 795 (39%) unique proteins, while that of Sup had 317 (15.5%) unique proteins. EVs and Sup shared nearly half of the proteins (927, 45.5%) (Fig. 4E). UB cell line derived EVs were found to carry large number of proteins involved directly or indirectly in Wnt signaling (*n* = 13), vascular endothelial growth factor (VEGF) signaling (*n* = 5), transforming growth factor-Beta (TGF-beta) signaling (*n* = 9), platelet-derived growth factor (PDGF) signaling (*n* = 6), Notch signaling (*n* = 3), integrin signaling (*n* = 24), Hedgehog (Hh) signaling (*n* = 3), fibroblast growth factor (FGF) signaling (*n* = 4), epidermal growth factor (EGF) signaling (*n* = 9), cytoskeleton regulation by Rho GTPase signaling (*n* = 8) and Cadherin signaling (*n* = 8) pathways (Fig. 4F). However, Sup contained very few or no proteins involved in different signaling pathways compared to that of EVs, such as Wnt signaling (*n* = 3), VEGF signaling (*n* = 0), TGF-beta signaling (*n* = 1), PDGF signaling (*n* = 1), Notch signaling (*n* = 1), integrin signaling (*n* = 1), Hh signaling (*n* = 0), FGF signaling (*n* = 1), EGF signaling (*n* = 1), cytoskeleton regulation by Rho GTPase signaling (*n* = 1) and Cadherin signaling (*n* = 2) pathways (Fig. 4F). The lists of proteins involved in different signaling pathways are presented in supplementary tables 4 and 5.

### Tracking of EVs during nephron formation

**Fig. 5:**
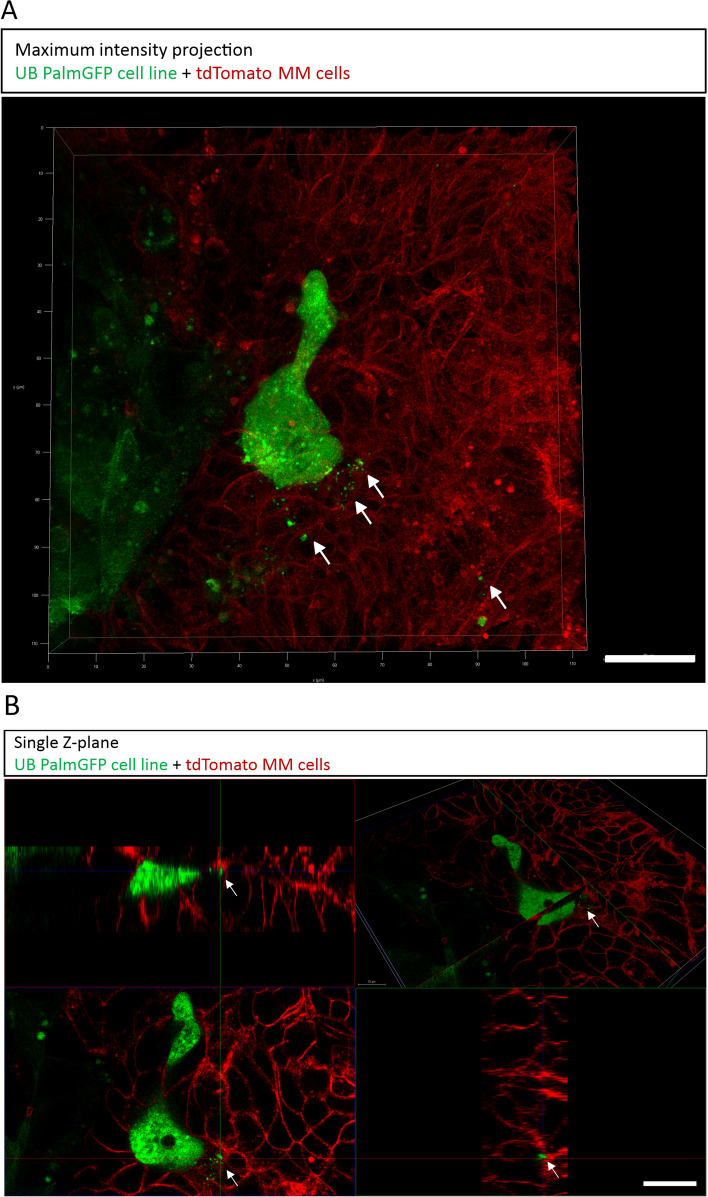
Tracking of EVs during nephron formation. **A** Confocal microscopy-based maximum intensity projection of a Z-stack image during early-stage nephrogenesis at day 2–3 showing UB PalmGFP Cell line EVs secreted inside the nephron forming zone. Arrows point to EVs. Scale bar: 20 μm. **B** Panel of single Z-plane confocal images showing EVs during nephron formation with different view planes. Arrows point to EVs. Scale bars: 20 μm

We further performed the tracking of EVs during nephrogenesis. For that, we generated UB cell line that stably expressed palmitoylated green fluorescent protein (PalmGFP) (UB PalmGFP cell line). Organoids were generated by the aggregation of 30,000 UB PalmGFP cell line cells and 50,000 tdTomato MM cells. E11.5 kidney MM cells expressing tdTomato were isolated from the mTmG mice. Kidney organoids were live imaged at early-stage nephrogenesis (day 2–3) in culture with Leica Sp8 Falcon Microscope. Results revealed that UB PalmGFP cell line actively secreted GFP tagged EVs during nephron formation. GFP tagged EVs were detected within the nephron formation zone as pointed with arrows (Fig. 5A). For the detailed analysis, organoid was imaged with side-view planes (Z-planes), which confirmed the presence of GFP tagged EVs inside the nephron formation zone (Fig. 5B). Our results suggest that EVs are actively secreted by the UB cell line during nephron formation.

### Influence of EVs on MM cells during nephrogenesis

**Fig. 6:**
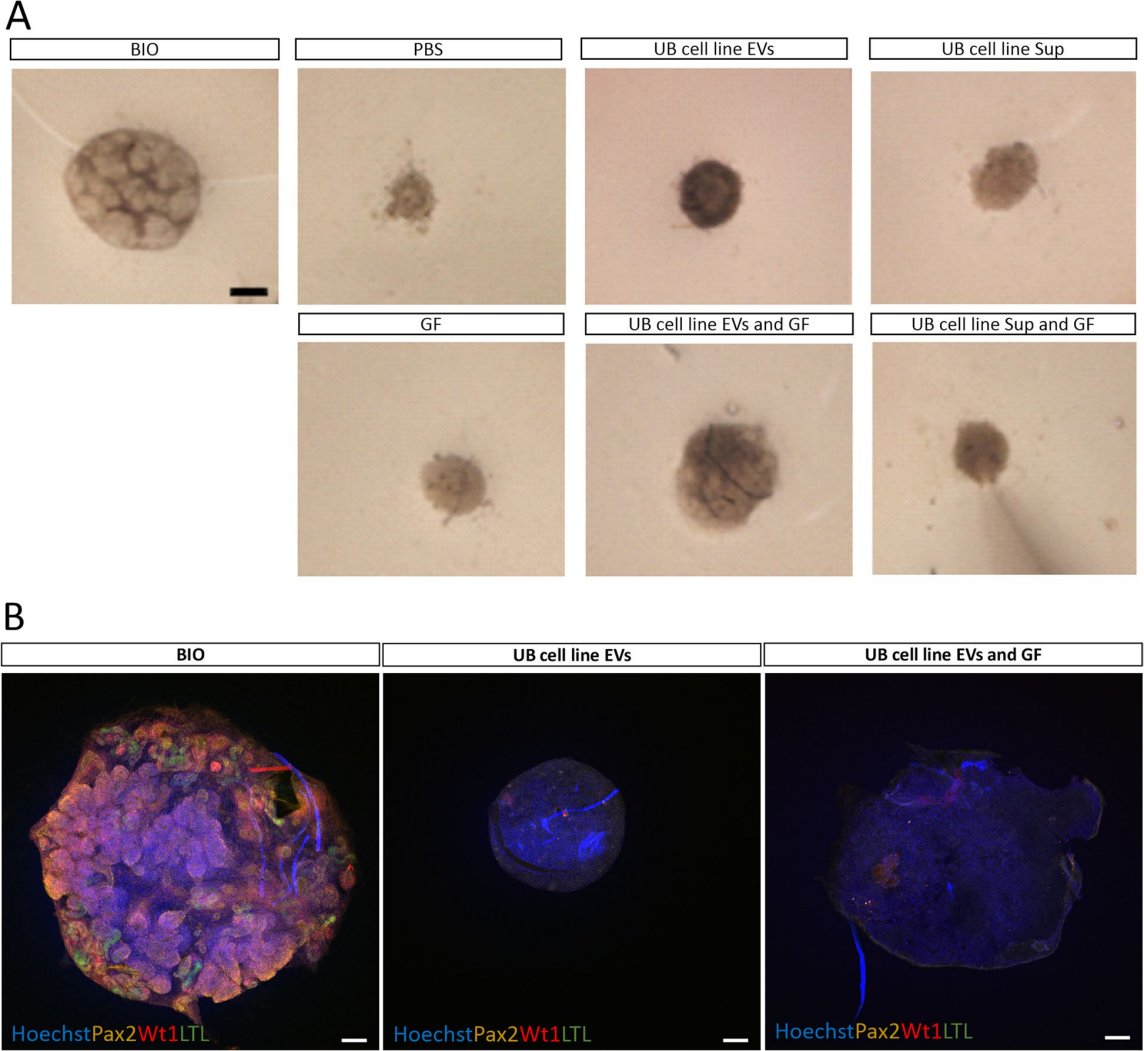
Influence of EVs on MM cells during nephrogenesis. **A** Bright field images of MM cell organoids with different treatments. Bright field images were captured at day 3. Scale bar: 250 µm. **B** Immunofluorescence confocal imaging (day 5) of cultured MM cell organoids with different treatments. Nephrogenesis was confirmed by staining the nephrons with markers: Pax2—proximal and distal tubules, Wt1—glomeruli, LTL—proximal tubules, Hoechst – nuclei. Scale bars: 100 µm

We investigated the function of EVs in nephrogenesis by different in vitro assays. MM cells from mouse kidney (E11.5) were treated with the UB cell line derived EVs (5 µg EV proteins), EV-free Sup and GF. Treated MM cells were centrifuged and then incubated at 37 °C overnight. MM cell organoids were transferred to the Trowel organ system for the growth. Our results revealed that only 6-bromoindirubin-3'-oxime (BIO) treated organoid (positive control) progressed to full-blown nephrogenesis, and none of the other organoids treated with PBS, GF (Bmp7 and Fgf2), UB cell line EVs, UB cell line EVs combined with GF, UB cell line Sup and UB cell line Sup combined with GF proceeded to nephrogenesis as shown by bright field imaging at day 3 of organoid culturing (Fig. 6A). Interestingly, the follow up study of organoids at day 5 showed that MM cell organoids treated with UB cell line EVs and UB cell line EVs combined with GF did not degenerate as quickly as the rest of organoids as shown with confocal imaging of organoids at day 5 (Fig. 6B). Confocal images showed successful nephrogenesis in organoid treated with BIO, but no nephron formation was observed in the organoids treated with UB cell line EVs and UB cell line EVs combined with GF as confirmed by staining with the markers of nephron (Pax2—proximal and distal tubule, Wt1—glomerulus, LTL—proximal tubule) (Fig. 6B). When a lower EV concentration (2 µg) was tested, organoids degenerated even faster (Supplementary Fig. 4). The results suggested that UB cell line derived EVs did not induce nephrogenesis in MM cells, although EVs prevented the quick degeneration of MM cell organoids compared to other treatments.

### Effect of EVs on the survival and nephrogenesis-competency of MM cells

**Fig. 7:**
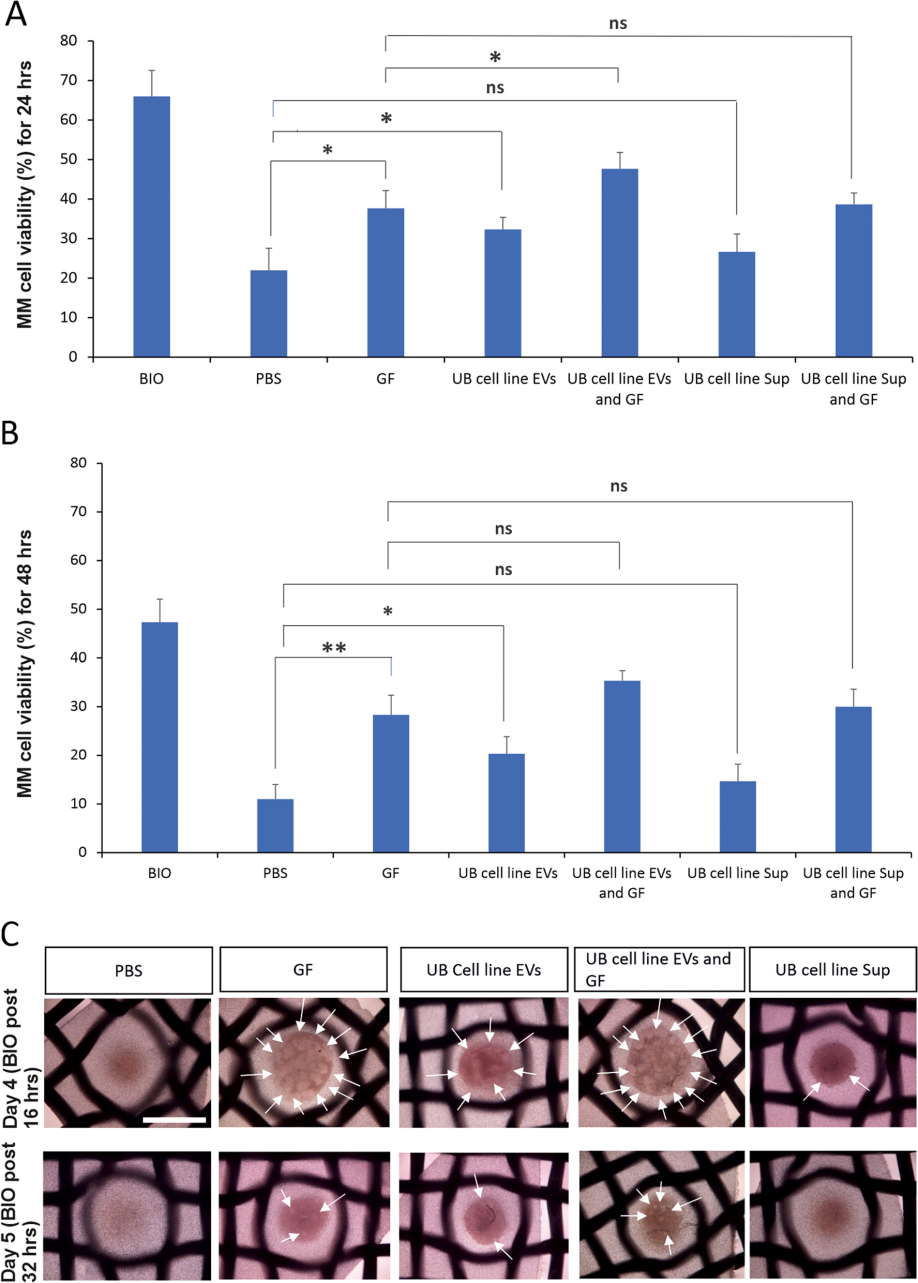
Impact of UB cell line derived EVs on the viability and nephrogenesis-competency of MM cells. **A-B** Graphs indicating the viability (%) of treated MM cells cultured in different conditions for 24 and 48 h. Statistical difference was determined using Student´s two-tailed t test. Levels of significance were described as: *P* > 0.05 (ns), *P* ≤ 0.05 (*), *P* ≤ 0.01 (**) and *P* ≤ 0.001 (***). Comprehensive statistical analysis of data was also performed by One-Way ANOVA with multiple comparison test (Supplementary tables 1 and 2). **C** MM cell organoid imaging (bright field) at day 4 (BIO added post 16 h of monolayer culture) and day 5 (BIO added post 32 h of monolayer culture). Developing nephron tubules are shown with arrows. Scale bar: 1 mm

The impact of EVs on the survival of MM cells was studied in MM cell organoids treated and cultured with the same protocol shown in Fig. 6A. The results revealed that MM cells treated with BIO exhibited the highest viability than any other conditions after 24 and 48 h in culture. Our data showed that GF (Fgf2 and Bmp7) significantly increased the viability of MM cells compared to the MM cells treated with PBS. The viability of MM cell organoid was also significantly enhanced by UB cell line derived EVs in comparison to the PBS treated MM cells, for 24 and 48 h. Moreover, UB cell line EVs in combination with GF significantly increased the viability of MM cells compared to the MM cells treated with GF for 24 h, but not for 48 h. Nevertheless, the viability of MM cells was not significantly increased by the addition of UB cell line Sup and UB cell line Sup with GF compared to the MM cells with PBS and MM cells with GF, respectively (Fig. 7A-B). Furthermore, UB cell line EVs combined with GF contributed to the best survival of MM cell organoid, although still lower than that of BIO. The results proposed that UB cell line EVs and GF maintain the viability of MM cells during nephron formation.

In the next experiment, the effect of EVs on the nephrogenesis-competency of MM cells was investigated in the presence of BIO. In this assay, kidney MM cells were monolayer-cultured under different treatments for 16 h. Here, the assay was divided in two sets: BIO-post 16 h and BIO-post 32 h. In BIO-post 16h, trypsinization of treated MM cells was performed after 16 h of monolayer culture, followed by centrifugation in Eppendorf tube, induction with BIO (3.5 ng/ml) and incubation at 37 °C overnight. MM cell organoids were transferred into Trowel organ culture for the formation and development of nephrons. The intensity of induced nephrogenesis was estimated by the number of newly forming nephrons as represented by the arrows in the figure (Fig. 7C). The results showed that MM cells maintained their competency for nephrogenesis in the presence of GF, UB cell line EVs and UB cell line EVs combined with GF, but not in the presence of PBS and UB cell line Sup. Bright field imaging of MM cell organoids was done at day 4 of monolayer culturing (Fig. 7C).

In BIO-post 32 h, induction of treated MM cells was done with BIO after 32 h of cell culturing by following the method applied to BIO-post 16 h set. The intensity of induced nephrogenesis was estimated by the number of newly forming nephrons as represented by the arrows in the figure (Fig. 7C). The results revealed that fewer MM cells maintained the competency for the formation of nephrons in the presence of GF, UB cell line EVs and UB cell line EVs combined with GF, but no MM cells maintained the competency in the presence of PBS and UB cell line Sup for formation of nephrons. Furthermore, MM cell treatment with the combination of EVs and GF maintained the highest competency than any other treated conditions. Bright-field imaging of MM cell organoids was done at day 5 of monolayer culturing (Fig. 7C). Our result indicated that the UB cell line derived EVs promote both the survival and nephrogenesis-competency of MM tissues during nephrogenesis.

### Genetic blocking of EVs

**Fig. 8:**
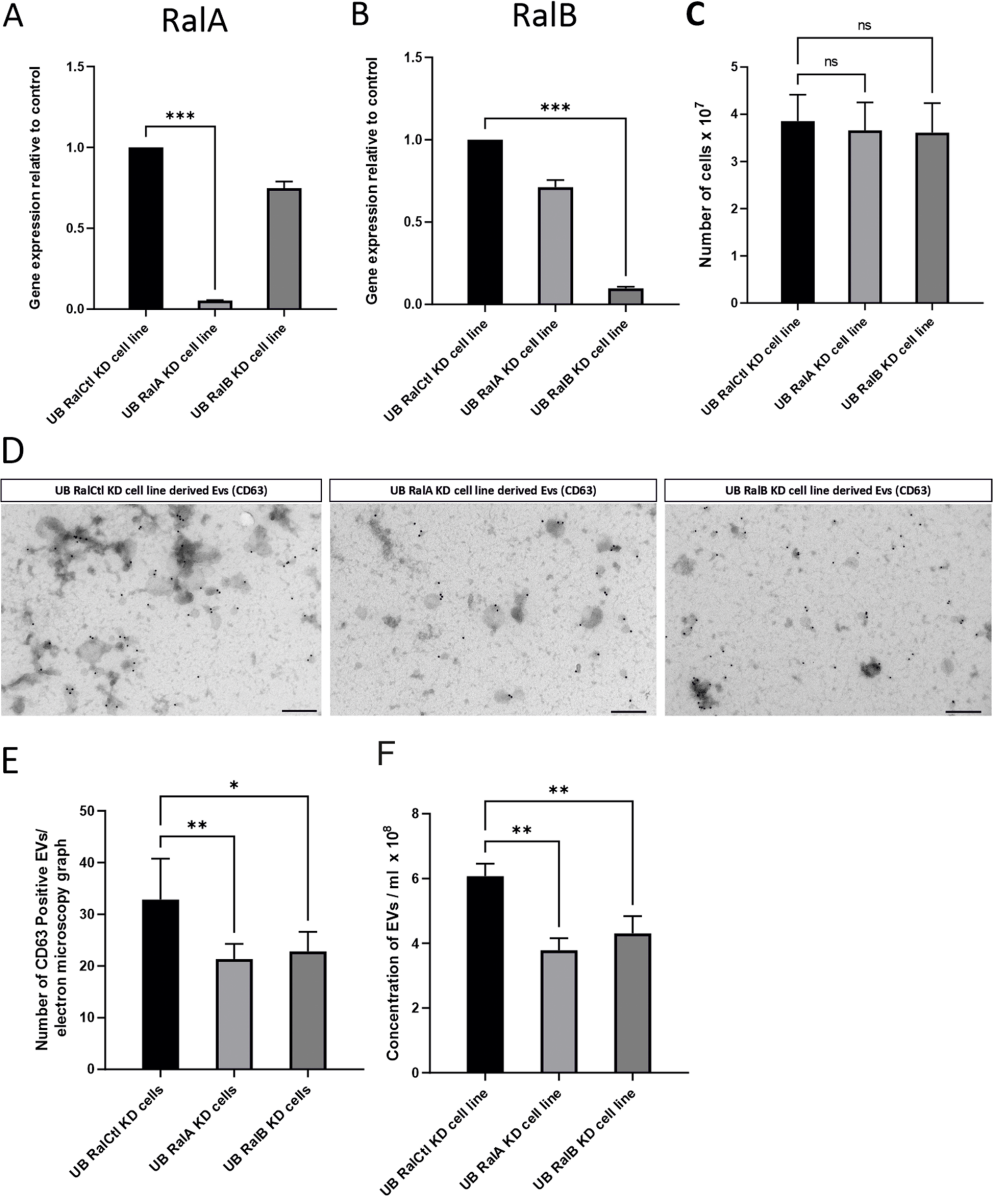
EV blocking by the KD of RalA and RalB gene expression. **A** Graph of qPCR result showing RalA gene expression relative to control in UB RalA KD cell line, UB RalB KD cell line and UB RalCtl KD cell line (*n* = 3). **B** Graph of qPCR result showing RalB gene expression in UB RalA KD cell line, UB RalB KD cell line and UB RalCtl KD cell line (*n* = 3). **C** Number of cells of the UB RalCtl KD cell line, the UB RalA KD cell line and the UB RalB KD cell line in four 14.5 cm cell culture dishes (*n* = 3). **D** Immunoelectron microscopy of EVs with anti CD63 antibody (*n* = 3). **E** Counting of CD63 positive EVs (*n* = 3). **F** Concentrations of EVs measured by NTA (*n* = 3). Statistical analysis of data was performed by One-Way ANOVA with multiple comparison test. Level of significance was shown as: *P* > 0.05 (ns), *P* ≤ 0.05 (*), *P* ≤ 0.01 (**) and *P* ≤ 0.001 (***)

In our study, the secretion of EVs was blocked by the knockdown (KD) of expression of RalA, RalB genes using shRNA. RalA and RalB genes modulate the biogenesis and the secretion of EVs. In our study, the stably transfected cells were termed as UB RalA KD cell line, UB RalB KD cell line and UB RalCtl KD cell line (scrambled shRNA). Our qPCR data showed approximately 95% KD of RalA gene expression and almost 90% KD of RalB gene expression in UB RalA KD cell line and UB RalB KD cell line, respectively. Interestingly, UB RalA KD cell line had some impact on RalB gene expression (nearly 25% decrease), whereas UB RalB KD cells had the similar effect over RalA gene expression (nearly 29% decrease) (Fig. 8A-B). The results showed that the proliferation of UB RalA KD cell line and UB RalB KD cell line was not affected by the KD of RalA and RalB gene expression, respectively, compared to that of UB RalCtl KD cell line. Cells were counted after the collection of cultured media and results revealed no significant difference among the cell numbers in all three types of cells (UB RalA KD cell line, UB RalB KD cell line and UB RalCtl KD cell line) (Fig. 8C). Subsequently, immunoelectron microscopy was performed to count the number of EVs isolated from different cell lines. Results showed higher number of CD63 positive EVs in the EV sample isolated from UB RalCtl KD cell line compared to that of UB RalA KD cell line and UB RalB KD cell line (Fig. 8D). Image J based EV counting analysis showed that the KD of RalA and RalB gene expression significantly blocked the secretion of EVs. In electron microscopy graph-based analysis (number of CD63 Positive EVs/ electron microscopy graph), approximately 21 (mean) CD63 positive EVs were detected in UB RalA KD cell line derived EV sample and approximately 23 (mean) CD63 positive EVs were detected in UB RalB KD cell line derived EV sample compared to that of 34 (mean) (approximately) in UB RalCtl KD cell line derived EV sample (Fig. 8E). Furthermore, NTA was performed to measure the number/ concentration of isolated EVs. As revealed by NTA, UB RalA KD cell line secreted approximately 3.78 × 10^8^ EVs/ ml, while UB RalB KD cell line secreted almost 4.30 × 10^8^ EVs/ ml, compared to 6.07 × 10^8^ EVs/ ml by UB RalCtl KD cell line, showing that approximately 38% and 29% EVs were significantly blocked in UB RalA KD cell line and UB RalB KD cell line, respectively (Fig. 8F). Our results suggest that the KD of RalA and RalB gene expression in UB cell line inhibit the secretion of EVs.

### Impact of EV blocking on nephrogenesis

**Fig. 9:**
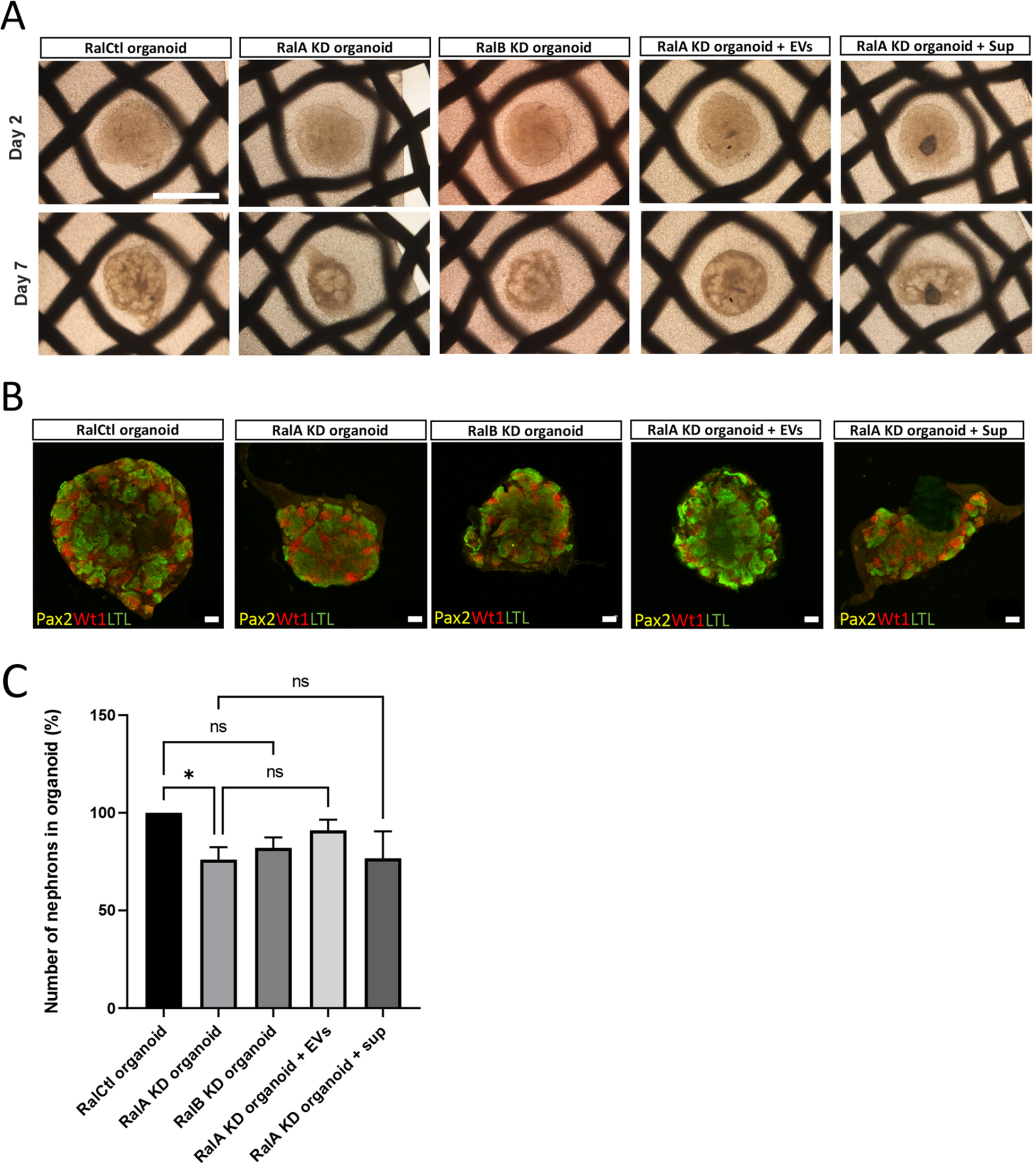
Effect of EV blocking on nephron formation. **A** Imaging (bright field) of organoid development at day 2 and day 7 in Trowel organ culture. Scale bar: 1 mm. **B** Confocal images of different organoids stained with markers of nephron (Pax2—proximal and distal tubules, Wt1—glomeruli, LTL—proximal tubules). Scale bars: 100 µm. **C** Quantification of the number of nephrons in organoids (*n* = 3). Statistical difference of data sets was determined using One-Way ANOVA and the significance was shown as: *P* > 0.05 (ns), *P* ≤ 0.05 (*), *P* ≤ 0.01 (**) and *P* ≤ 0.001 (***)

Our previous experiment confirmed the significant blocking of EVs via the KD of RalA and RalB gene expression in UB cell line. The impact of EV blocking was studied in nephrogenesis, where the kidney organoids were generated by the aggregation of 50,000 MM cells with 30,000 cells of UB RalA KD cell line, UB RalB KD cell line and UB RalCtl KD cell line. Organoids were cultured overnight in Eppendorf tube at 37 °C and then transferred into Trowel organ culture. Imaging (bright field) at day 2 showed that all different organoids had almost the equal size, but later at day 7 organoids generated by the aggregation of MM cells with UB RalA KD cell line and UB RalB KD cell line, developed to smaller size compared to RalCtl organoids (Fig. 9A-B). Nevertheless, the treatment of RalA KD organoids with the UB cell line EVs (5 μg EV protein) improved the size of organoid (RalA KD organoid + EVs) toward the normal size. The treatment of RalA KD organoids with the UB cell line Sup (5 μg EV Sup) had only a minor impact on the organoid development (RalA KD organoid + Sup) (Fig. 9A-B). In immunofluorescence assay, organoids were stained with nephron markers (Pax2, Wt1 and LTL) to measure the impact of EV blocking on nephrogenesis (Fig. 9B). Result showed that due to the blocking of EVs, the number of nephrons was decreased to approximately 76% and 82% in RalA KD and RalB KD organoids, respectively, compared to 100% in RalCtl organoid. The decrease in the number of nephrons in RalA KD organoids was statistically significant, whereas that of RalB KD organoid was not significant (Fig. 9B-C). Moreover, the addition of UB cell line EVs (5 μg EV protein) to RalA KD organoid exerted a tendency toward improving the nephrogenesis (RalA KD organoid + EVs), but this effect was not significant statistically. The addition of UB cell line sup (5 μg Sup protein) to the RalA KD organoid did not exert any significant improvement in nephrogenesis (RalA KD organoid + Sup) (Fig. 9B-C). These experiments indicate that EVs are the integral part of nephrogenesis, which promote the process of nephron formation and their blocking impair nephrogenesis.

## Discussion

Nephron formation takes place via the bidirectional and continuous communication of UB and MM. Many signaling molecules are exchanged in this interaction in embryonic mouse kidney. How the UB and MM cells interact via signaling molecules is not completely understood. The signaling molecules important for nephrogenesis include Wnts, cell growth factors and other inducer molecules [10–12]. Our study investigated the signaling interaction via secreted EVs during nephrogenesis using a mouse model. EVs carry cargo of proteins, nucleic acids and other active molecules to the target recipient cells to perform a variety of functions in organ development and pathogenesis. Investigating the role of EVs in nephrogenesis is challenging due to the limitations with the short time cell culturing of E11.5 mouse kidney cells and the small cell number obtained from kidney E11.5 UB and MM tissues. To solve this issue, our study focused on generating a novel in vitro flexible model of nephrogenesis, which allows efficient genetic modification, as well as comprehensive characterization of EVs, tracking of EVs and genetic blocking of EV production during nephrogenesis.

In the current study, we utilized UB cell line to generate an in vitro flexible model of nephrogenesis by aggregating it with mouse E11.5 MM cells in the presence of GF (Fgf2 and Bmp7). This model formed segmented nephrons demonstrated by the immunofluorescence of nephron organoids with anti Pax2, anti Wt1 and anti LTL antibodies. Also, this model allowed efficient cell culturing, genetic manipulation, characterization of EVs, tracking of EVs and studying the functions of EVs in nephrogenesis in an easy way compared to the available models. The previously developed in vivo, in vitro and mouse embryonic stem cell models are laborious, expensive and time-consuming, and have limitations with cell culturing, preventing comprehensive EV research [12, 14–16]. In the last decade, mouse embryonic stem cells were utilized to generate kidney organoid as an alternative to in vivo nephrogenesis, but those models also could not easily be applied to EV research since they are not suited for long-term cell cultures [13, 14, 17, 18]. Thus, we propose the UB cell line based in vitro flexible model as a model system for nephrogenesis and EV research.

The current study aimed to determine the suitable ratios of UB cell line and MM cells needed for efficient nephrogenesis. Our results showed that minimum 5,000 UB cell line cells are required to start nephron formation in 50,000 MM cells. Most efficient nephrogenesis was achieved, when 30,000 UB cell line cells were mixed with 50,000 MM cells. Results of this study are in agreement with the previous in vivo and in vitro reports for inducing nephrons in MM cells, where suitable ratios of primary kidney E11.5 MM and UB cells for inducing nephrons were proposed [19].

After the development of the flexible model of nephrogenesis, this study focused on the characterization of UB cell line derived EVs. Results showed that UB cell line secreted EVs are round with the size range of 50—150 nm diameter, and they express the standard markers of EVs, including CD63, CD9, TSG101 and CD81. Also, these results agree with the results obtained from E11.5 UB cell EVs, where EVs had a round shape with the size diameter of 30—150 nm and carried CD63 as a standard EV marker. Our results also agree with the previously published studies regarding the characterization and morphology of EVs [1, 2, 20].

In this study, UB cell line actively secreted EVs to the nephron formation zone, suggesting that EV secretion happens during nephrogenesis (Fig. 5A-B). This result was further solidified by showing that E11.5 UB and MM cells secreted EVs (Fig. 1A-D). Previous studies did not directly track the secretion of EVs during active organogenesis, although during metastatic cancer EVs were actively secreted and tracked in immune cells [21].

In our study, results from different assays revealed that the treatment of MM cells with UB cell line EVs did not induce nephrogenesis, although EVs significantly increased the viability and nephrogenesis-competency of MM cells. The increased viability and nephrogenesis-competency of MM cells may be attributed to the presence of large number of EV proteins involved directly or indirectly in Wnt signaling, VEGF signaling, TGF-beta signaling, PDGF signaling, Notch signaling, integrin signaling, Hh signaling, FGF signaling, EGF signaling, cytoskeleton regulation by Rho GTPase signaling and Cadherin signaling. These proteins were absent in the UB cell line Sup, which is depleted of EVs, and Sup did not significantly enhance the viability and nephrogenesis-competency of MM cells. Previous studies were found partly or wholly in line with our results. Interestingly, in a previous study EVs were shown to regulate embryonic tooth development. EVs reciprocally induced the differentiation of tissues and matrix synthesis. For example, epithelium derived EVs evoked mesenchymal cells to synthesize dentin sialoprotein and to ultimately proceed to mineralization, while mesenchymal cell secreted EVs stimulated epithelium to synthesize amelogenin and ameloblastin [22]. Salivary gland formation occurs due to reciprocal communication of epithelium and mesenchyme. Mesenchyme secreted exosomes containing miRNA were transported into epithelium tissue, where they regulated organ morphogenesis [23]. Moreover, human dermal papilla derived exosomes contributed to the development and growth of hairs [24].

The biogenesis and secretion of EVs are complicated mechanisms and many pathways are involved in these mechanisms. In our study, we partially blocked the biogenesis/secretion of EVs by the KD of RalA and RalB genes. Interestingly, this blocking partially impaired nephrogenesis suggesting that EVs support nephrogenesis. This finding was further solidified by the results that the addition of UB cell line EVs rescued the impaired nephrogenesis to some extent. Also, this finding is in agreement with the results obtained when the MM cells were treated with the UB cell line EVs, wherein EVs supported nephrogenesis by increasing the viability and nephrogenesis-competency of MM cells (Fig. 7A-C). This agreed with previously published results, wherein the blocking of exosomes disturbed tooth organogenesis in Rab27a mutant mice [22]. On one hand, the RalA and RalB genes are important in the biogenesis of EVs, on the other hand, the RalA and RalB genes were expressed in the developing embryos and embryonic organs, such as gonads and adrenal gland [25, 26].

EVs are complex entities involved in cell–cell communication. Studying the role of EVs in organogenesis/ nephrogenesis, where multiple layers of cell interactions are involved is even more challenging. Previous literature showed that the interaction of UB and MM is bidirectional, and this interaction is a continuous process until the advanced stages of nephrogenesis. In this process UB sends early-stage signals to MM that stimulate MM for differentiation. In return, MM sends back signals to the UB for branching morphogenesis. At the next phase, UB again provides signaling in the form of Wnts that stimulate MM for differentiating into pre-tubular aggregates and renal vesicles. During this process UB is further stimulated for branching by the signals received from MM, and so on the continuous bidirectional communication takes place during nephrogenesis [10, 11, 14, 15]. In in vitro experiments (Figs. 6 and 7), UB cell line derived EVs did not induce nephrogenesis in MM cells but significantly contributed to the survival and nephrogenesis-competency of MM cells, which reveals one of the roles of UB cell EVs toward MM cells prior/during nephrogenesis. These results were obtained from experiments where only a one-way EV signaling was established from the UB cell line to the MM cells by adding EVs to the MM cells. The results suggest that UB cell EVs are needed by the MM cells to stay alive and undergo nephrogenesis. Importantly, in another experiment (Fig. 9), EV inhibition in UB cell line partially impaired the ability of UB cell line to induce nephrogenesis. In this experiment, the UB cell EVs were continuously and intrinsically blocked during the ongoing nephrogenesis process. Therefore, the nephrogenesis was continuously impaired by the EV blocking. Overall, both experiments confirm the presence of EV-based communication in nephrogenesis. However, how critical is the EV-based communication compared to direct cell–cell interactions in nephrogenesis is not understood yet. The direct cell (UB)-cell (MM) contact during nephrogenesis is believed to be the main factor that induces full blown nephrogenesis [10–12, 27]. Also, our data as shown in Fig. 2A-C are in line with these studies. However, during nephrogenesis, in addition to direct cell–cell contact based communication, intercellular communications also happen by secreted entities/factors, which partly contribute to cell–cell communication and nephrogenesis. EVs are secreted by the cells, and they carry different secreted factors. In our study, EVs from the nephrogenesis-inducting UB cell line helped the MM cells to survive and nephrogenesis-competent suggesting that UB cell EVs support nephrogenesis (Figs. 6 and 7). The results also suggest that survival and nephrogenesis-competency of MM cells by the EVs are happening during nephrogenesis in addition to the induction of nephrogenesis by the cell–cell contact. Based on the revealed results, the EVs were observed to be one part of communication, that is happening during nephrogenesis. Nephrogenesis/ organogenesis is a complex sequential process where many layers of communication happen including direct cell–cell contact, secreted factors and EVs. During this communication, MM tissues are sequentially differentiated into cap mesenchyme, pre-tubular aggregates, renal vesicles, comma-shaped bodies, S-shaped bodies and mature nephrons [10, 11, 13]. The results from the current study present novel data showing that EV-based communication also happens in nephrogenesis in addition to direct cell (UB)-cell (MM) contact/ communication. Interestingly, a few studies showed EVs to be a part of cell–cell contact. For example, cellular protrusions may act as a road to transport EVs into target cells, which suggest the direct relationship of EVs and cell–cell contact by protrusions [28]. Filopodia are extended to the neighboring cells to form EVs to assist developmental processes, such as cell migration, cell behavior and cell adhesion [29–31]. EVs were seen traveling along the cell protrusions to the neighboring cells [32]. The current study advanced the understanding of complex nephrogenesis process and described EV-based communication as one part of communications in nephrogenesis. This study encourages cell line-based organoid models for organogenesis research. Cell line-based organoid models are cost effective, easy to handle and allow efficient genetic manipulation compared to the currently available models for organogenesis.

## Conclusions

Our study developed a flexible and easy-to-use model for nephrogenesis based on the organoid formed by UB cell line and mouse kidney MM cells. This model made it possible to study the fundamental role of EVs in nephrogenesis, which was not possible with the previous in vivo, in vitro and mouse embryonic stem cell organoid models. The current study confirmed the presence of EV-based communication during nephrogenesis. This EV-based communication facilitated nephrogenesis by increasing the viability and nephrogenesis-competency of nephron forming cells (MM cells) rather than directly inducing nephrogenesis. Interestingly, when the secretion of UB cell line EVs was blocked by the KD of RalA and RalB, nephrogenesis was impaired, but the addition of UB cell line EVs rescued the impaired nephrogenesis to some extent. EVs alone without direct UB cell line-MM cell communication did not induce nephrogenesis, suggesting that direct UB and MM interaction is necessary for nephrogenesis. Altogether, our study suggests that EV-based communication supports nephrogenesis, but the exact function of EVs is still unknown. This study opens new avenues for the link between EVs and organogenesis, and is likely to facilitate future studies to understand the mechanisms of organ generation. Further studies are also needed to clarify the role of EVs in the induction of nephrogenesis.

## Materials and methods

### Animal licenses and ethical permissions

In our study, animal care and procedures were performed according to the protocols of the Finnish national legislation for laboratory animals, EU Directive 86/609/EEC and European Convention for the protection of vertebrate animal used for experimental and other scientific purposes (ETS 123). Experimental use of wildtype CD-1 (CD1) and floxed Rosa26 GFP mT/mG reporter (mTmG) mice were authorized by the National Animal Experiment Board Finland (ELLA) and the Laboratory Animal Center of University of Oulu. Licenses included CD1 mouse license: 17/2021 and mTmG mouse license: 12/2018. The CD1 and mTmG mice have been described previously [12, 33].

### Dissection of mouse kidneys and preparation of primary kidney cells

E11.5 mouse embryonic kidneys were dissected from CD1 or mTmG mice according to the methods used in the previous study [12]. E11.5 kidneys were used to isolate UB and MM tissues. The kidneys were treated with trypsin (2.25%) and pancreatin (0.125%) (Sigma Aldrich, United States) for 30 s at room temperature. Kidneys were transferred into DMEM high glucose medium (Cat # 11965092, Gibco, ThermoFisher Scientific, United States) supplemented with 10% fetal bovine serum (FBS) and 1% penicillin/streptomycin. MM and UB kidney tissues were separated from one another using syringe. Separated MM and UB tissues were used for subsequent experiments, such as cell culturing, organoid generation and EV isolation. MM tissues were incubated with 40 μl of collagenase type 3 (Cat # CLS-3/LS004180, Worthington Biochemical Corporation, United States) in 280 μl of physiological buffer (137 mM NaCl, 2.2 mM CaCl_2_, 5.6 mM KCl, 2.5 mM glucose, 1.2 mM MgCl_2_ × 6H_2_O and 10 mM HEPES) for 20 min at 37 °C. MM tissues were triturated with pipetting for 3–5 min until they were dissociated into single cells. DMEM high glucose medium (supplemented with 10% FBS and 1% penicillin/streptomycin) was added to MM cells for the inhibition of collagenase activity. Cell suspension was centrifuged at 1400 × g for 4 min to pellet MM cells, which was used for subsequent cell culturing and generation of nephron organoid.

### Isolation of EVs from primary kidney UB and MM tissues and cells

E11.5 MM cells and E11.5 UB tissues were cultured in DMEM high glucose medium containing EVs-depleted 10% FBS (Cat # F7524, Sigma-Aldrich) and 1% penicillin/streptomycin, for 48 h in 48-well cell culture plates (Corning, United States). Prior to the use in the cell culture, the medium (10% FBS in DMEM high glucose medium) was processed by performing two ultracentrifugation at 120,000 × g overnight at 4 °C to deplete the FBS EVs from the medium. MM cells were provided with the growth factors (GF): Fgf2 (100 ng/ml) (Cat # 100-18C, PeproTech, London, United Kingdom) and Bmp7 (50 ng/ml) (Cat # 120-03P, PeproTech, London, United Kingdom) in culturing. UB tissues were grown in recombinant human GDNF (100 ng/ml) (Cat # 212-GD-010/CF, R&D Systems, United States) in the medium. Media from cultured MM and UB were collected continuously till the volume of collected media reached to 5—10 ml. Cultured media were processed to isolate EVs. Media were centrifuged at 300 × g for 20 min to remove the dead cells. In the next step, cell debris was discarded by centrifugation at 2,000 × g for 20 min and at 3,000 × g for 20 min at 4 °C. Supernatants were filtered through 0.8 μm filters (Cat # 16,592, Minisart® Syringe Filter, Sartorius, Germany). Filtered supernatants were ultracentrifuged at 100,000 × g overnight at 4 °C. EV pellets were resuspended with phosphate buffered saline without Calcium and Magnesium (PBS).

### UB derived cell line culturing and isolation of EVs

#### UB derived cell line culture

The E11.5 UB derived cell line (UB cell line), which has been immortalized, was gifted by Barasch´s lab, Columbia University, United States [27]. UB cell line was cultured in DMEM/F-12 medium (Cat # 10565018, DMEM/F-12 GlutaMAX, Gibco, ThermoFisher Scientific, United States) supplemented with 10% FBS and 1% penicillin/streptomycin. Cells were incubated in 5% CO_2_ at 37 °C.

### Isolation of EVs from UB cell line

UB cell line was cultured and passaged into four bigger cell culture plates (14.5 cm) (Ref. 639,160, Greiner BIO-ONE, Austria). Once the cells reached to 70–80% confluency, they were washed three times with PBS, and provided with DMEM/F-12 medium (20 ml) with 1% penicillin/streptomycin and without FBS. Cells in plates were cultured for 24 h, and medium (4 × 20 ml = 80 ml) containing the EVs was collected and processed according to the protocol used for the primary kidney cells. The cell counting assay showed that the plates contain approximately 3.7 × 10^7^ cells after the isolation of cultured medium. Cell counting was performed using TC20 Automated Cell Counter (Bio-Rad, United States). For the ultracentrifugation (100,000 × g), the medium (80 ml) was processed in 3 ultracentrifugation tubes. After ultracentrifugation, EV pellet in a tube was resuspended with 40 μl PBS (3 × 40 μl PBS = 120 μl PBS containing EVs). Also, this protocol was used for the isolation of EVs from UB cell lines with the KD of RalA, RalB and Ral scrambled (RalControl) gene, where the equal number of cells were plated in four 14.5 cm cell culture plates for each type of cells (RalA, RalB and RalControl cell line). After the collection of media, cells were counted using TC20 Automated Cell Counter. The blocking of EVs due to the KD of RalA and RalB gene was confirmed by measuring the number of isolated EVs (RalA, RalB and RalControl cell line derived EVs) using NTA and immunoelectron microscopy. For the characterization of UB cell line derived EVs, in Western blot experiments, supernatant after filtration and before ultracentrifugation (100,000 × g) was used as a control and this was termed as conditioned medium (CM). Moreover, EVs depleted supernatant after 100,000 × g (ultracentrifugation) was used as a control and this supernatant was termed as Sup in different experiments, such as Western blot, proteomics, immunoelectron microscopy, MM cell treatment and organoid experiments as shown in the results. CM and Sup were always concentrated before using them in the experiments.

### Concentrating CM and Sup samples

To concentrate CM and Sup samples, Centricon Plus-70 centrifugal filters (Cat # UFC701008, Merck Millipore, Germany) were used. Centrifugation of 60 ml sample in filters was performed at 3500 × g for 45 min to collect (200 μl) concentrated CM and Sup in collection cup.

### NTA of EVs

NTA was performed to measure the size and concentration of EVs using NanoSight NS300 (Malvern Panalytical, United Kingdom). NanoSight NS300 coupled with laser (405 nm) was used for the operation. EV samples were diluted in 1:400 in MilliQ water and 1 ml diluted sample was loaded into NanoSight NS300. In the operation, EV samples were inspected by four-time eight interval video recording (60 s) with detection threshold level at 3 and camera level at 14. EV measurement data was calculated with the NTA software version 3.4.

### Immunolabelling and electron microscopy analysis of EVs

In immunoelectron microscopy assay, EVs were labelled with anti CD63 antibody (Cat # D263-3, MBL International Corporation, United States). EV sample (3 μl) was applied to Formvar copper grid for 20 min. EV sample on grid was blocked for 10 min using 1% Aurion bovine serum albumin (BSA) (Cat # 900.099, Aurion, Netherlands) in PBS. EVs were incubated with primary antibody (anti CD63) for 20 min. Primary antibody was diluted as 1:50 in Aurion BSA (0.1%) in PBS. Samples were incubated for 20 min with secondary antibody (anti rat) (Cat # 312–005- 003, Jackson ImmunoResearch, United Kingdom). Dilution (1:200) for secondary antibody was made in 0.1% Aurion BSA in PBS. Next, gold coupled protein A was applied to the EVs in grids for 20 min. Samples of EVs were fixed for 5 min using 1% glutaraldehyde in PBS. Sample incubation was performed for 5 min in 2% neutral uranyl acetate. In the next step, EVs were sequentially passed through 3 drops of 2% methylcellulose-uranyl acetate. EVs were imaged by Tecnai G2 Spirit TEM (FEI, Eindhoven, The Netherlands). QUEMESA camera (Olympus Soft Imaging Solutions, Germany) fitted in TEM was used for imaging. After imaging, EVs stained with anti CD63 antibody were counted in each electron microscopy graph using Image J software (Fiji) in the cases of inhibition of EVs by the KD of RalA and RalB.

### Immunolabelling of E11.5 kidney

Kidneys (E11.5) were fixed using 4% paraformaldehyde (PFA) and 2.5% sucrose in 0.1 M phosphate buffer. Next, kidneys were shifted to 2.3 M sucrose in PBS and then placed in 2% melted gelatin in 0.1 M phosphate buffer in an orientation where the interaction of UB and MM was clearly observable. Kidneys embedded in gelatin were frozen in liquid nitrogen followed by cutting solidified gelatin into ≤ 80 nm thick sections. Kidney sections were placed in grids on 2% melted gelatin overnight. Blocking of kidney section was done for 15 min using 1% Aurion BSA in PBS. Kidney sections were incubated with anti CD63 antibody, which was diluted as 1:50 in 0.1% Aurion BSA in PBS for 45 min. Kidney sections were then incubated with secondary antibody against anti CD63 antibody in 0.1% Aurion BSA in PBS for 30 min. Samples were treated for 30 min with gold coupled protein A diluted in 0.1% Aurion BSA in PBS. Kidney sections were treated with 1% glutaraldehyde in PBS for 5 min. Sections were then treated with 2% neutral uranyl acetate for 5 min. Afterwards, samples were passed through 3 drops of 2% methylcellulose-uranyl acetate on ice. Finally, samples were studied by Tecnai G2 Spirit TEM.

### Western blot of EV samples

The protein-based concentration of EVs, CM and Sup was measured using Pierce BCA Protein Kit (Cat # 23225, ThermoFisher Scientific) for bicinchoninic acid assay according to the protocols provided by the manufacturer. Samples (40 µg proteins) were separated on 12.5% sodium dodecyl sulphate polyacrylamide gel electrophoresis (SDS-PAGE) by applying 120 Volt (V) in running buffer for 90 min. In the next step, proteins were transferred to the nitrocellulose membranes by applying 110 V in transfer buffer for 70 min. Membrane blocking was done with 5% skimmed milk prepared in Tris-buffered saline containing 0.1% Tween 20 (1 × TBST). Incubation of membranes with primary antibodies was performed overnight at 4 °C. Primary antibodies included: TSG101 (SC-7964, Santa Cruz Biotechnology) with 1:200 dilution, CD81 Antibody (SC-166029, Santa Cruz Biotechnology) with 1:200 dilution and CD9 (Ab92726, Abcam) with 1:1000 dilution. Afterwards, membranes were incubated for 1 h with respective secondary antibodies (HRP conjugated antibodies) (Dako, Agilent Denmark).

### Mass spectrometry-based proteomics of EVs

Samples of UB cell line EVs and UB cell line supernatant (Sup) were prepared for mass spectrometry-based proteomics analysis. UB cell line EVs and UB cell line Sup were prepared from the UB cell line according to the protocol described in the sections of Materials and methods: Isolation of EVs from UB cell line and Concentrating CM and Sup samples. The cell counting assay showed that the plates contain approximately 3.7 × 10^7^ cells after the isolation of cultured medium. Protein sample volume corresponding to 100 μg of protein was mixed with three time volume of Milli-Q water, four time volume of methanol and one time volume of chloroform to precipitate the proteins. Protein precipitate was mixed with methanol. Protein precipitates were mixed with 4 × Laemmli sample buffer. Samples were reduced with 20 mM dithiothreitol (DTT), followed by alkylation of protein samples with 45 mM Iodoacetamide (IAA). Samples were loaded on 12% gel (Cat # 4561043, Bio-Rad, United States). Gels were fixed using fixation solution (50% ethanol, 10% acetic acid in Milli-Q water) for 30 min. Gel staining was done using SYPRO Ruby gel stain (Cat # S12000, ThermoFischer Scientific, United States) at room temperature overnight, followed by de-staining with 5% acetic acid in Milli-Q water. Bands (proteins) were cut under ultraviolet (UV) light and sent for liquid chromatography mass spectrometry (Proteomics and protein analysis core facility, Biocenter Oulu, University of Oulu). The experiment was performed in triplicate for both the UB cell line EVs and UB cell line Sup. Raw data from liquid chromatography mass spectrometry was analyzed by Proteome Discoverer (version 2.2, Thermo Scientific). Tandem mass spectrometry (MSMS) spectra from ion trap were processed with 0.6 Da (mass tolerance) and 0.02 Da (orbitrap data). Raw data analysis was done with the Swissport database for mouse with search setting: precursor mass tolerance (10 ppm), trypsin cleavage (up to 2 missed cleavages), oxidation with optional modification on methionine, carbamidomethyl with modification on cysteine, optional deamidation on glutamine and asparagine, and optional acetylated protein N-terminus. Percolator node was done by FDR 0.01. Detector (minora feature) was selected for quantification (label free) using retention time alignment for 2 min. Precursor intensity was standardized for the total peptide amounts. Peptide numbers were analyzed and counted from intensities of precursors and were then standardized to total number of peptides. Protein hits with 2 or more peptides were counted. Comparative analysis of EV and Sup proteins was done using Venny tool version 2.1 [34]. Only the common proteins detected in each replicate of EVs (triplicate) were considered for the subsequent bioinformatics analysis, and same was followed for the Sup samples. Further bioinformatics analysis was performed with PANTHER™ Version 17.0 [35].

### Generation and analysis of organoids

#### Generation of nephron organoids using UB cell line and E11.5 primary MM cells

UB cell line cells were aggregated with E11.5 dissociated MM cells to generate kidney organoid containing segmented nephrons. MM cells were derived from E11.5 CD1 or mTmG embryos. In these experiments, MM cells were provided with GF (50 ng/ml Bmp7 and 100 ng/ml Fgf2) before mixing them with UB cell line. Different ratios of UB cell line and E11.5 dissociated MM cells were used in different assays. To check whether UB cell line can induce MM cells to generate nephrons or not, 50,000 UB cell line cells were aggregated with 100,000 MM cells. For comparison with UB cell line, five E11.5 UB tissues were mixed with 100,000 MM cells for the induction of nephrogenesis. Moreover, to determine the optimal ratio for nephrogenesis, UB cell line and MM cells were mixed in different ratios: 2,500 UB cell line: 50,000 MM cells, 5,000 UB cell line: 50,000 MM cells, 10,000 UB cell line: 50,000 MM cells, 15,000 UB cell line: 50,000 MM cells, 20,000 UB cell line: 50,000 MM cells, 30,000 UB cell line: 50,000 MM cells, 40,000 UB cell line: 50,000 MM cells and 50,000 UB cell line: 30,000 MM cells. In the experiments with the KD of RalA and RalB gene, organoids were generated by using a ratio of 30,000 UB cell line cells and 50,000 MM cells. In the next step, cell mixtures for the organoids with the KD of RalA gene were provided with the UB cell line EVs (5 μg protein) or the UB cell line Sup (5 μg protein) before pelleting the cell mixtures in LoBind Eppendorf tubes. Afterwards, the cell mixtures were centrifuged, cultured, imaged and fixed according to the protocol described next. To track the secretion of EVs by UB cell line during nephrogenesis, 30,000 UB PalmGFP cell line cells were aggregated with 50,000 mTmG MM cells. Mixtures of UB cell line (or E11.5 UB tissues) and MM cells were centrifuged at 1400 × g for 20 min in LoBind Eppendorf tubes. Organoids were incubated overnight at 37 °C. Next day, organoids were transferred into Trowel type organ culture on 0.1 μm filter in DMEM high glucose media with 10% FBS and 1% penicillin/streptomycin. The medium was replaced with the fresh medium every second day and if necessary, the organoids were imaged with Olympus camera (Model # E-P1, Olympus, Japan) during their growth and development. Organoids were fixed with 4% PFA at day 7 of generation.

### Live imaging of organoids for tracking of EVs in real time

Organoids generated by the aggregation of 30,000 UB PalmGFP cell line cells and 50,000 mTmG MM cells were live imaged with Leica SP8 FALCON microscope (Leica Microsystems, Germany). Due to the fragility of organoids, they were subjected to live imaging at day 2 of generation.

### Immunofluorescence and imaging of organoids

Kidney organoids were fixed with 4% PFA for 30 min and washed three times with PBS. Organoids were blocked for 3 h with blocking buffer containing 1% BSA, 0.1% Triton X-100 (Cat # T8787, Sigma Aldrich, United States), 10% FBS and 10% goat serum in PBS at room temperature. Kidney organoids were incubated with primary antibodies overnight at 4 °C. Primary antibodies included anti Wt1 antibody with 1:200 dilution (Cat # 05–753, MerckMillipore, United States) and anti Pax2 antibody with 1:300 dilution (Cat # PRB 276P, Biolegend, United States) in blocking buffer. Anti Wt1 antibody stains glomerulus, whereas anti Pax2 antibody stains both distal and proximal tubule of nephron [14]. In the next step, organoids were incubated with Hoschst (1:1000 dilution) (Cat # 62249, ThermoFisher Scientific, United States) and respective secondary antibodies, including goat anti rabbit Alexa Fluor 647 (1:1000 dilution) (Cat# A21244, ThermoFisher Scientific, United States) and goat anti mouse Alexa Fluor 546 (1:1000 dilution) (Cat # A21123, ThermoFisher Scientific, United States). Also, another antibody, LTL Fluorescein (1:1000 dilution) (Cat # FL-1321, Vector Laboratories, United States) was used for staining proximal tubule of nephron. Finally, organoids were mounted using Immu Mount (ThermoScientific, United States). Organoid imaging was done using Zeiss LSM-780 confocal microscope (Zeiss, Germany). Nephron counting in organoids was done using Zeiss Zen software (blue edition) by applying the maximum intensity projection.

### Gene expression analysis by quantitative polymerase chain reaction

RNA from UB RalA KD cell line, UB RalB KD cell line and UB RalCtl KD cell line was extracted using RNeasy kit (Cat # 74106, Qiagen, Germany) by following the manufacturer´s guideline. Complementary DNA (cDNA) was prepared using 1 μg RNA according to the guideline of first strand cDNA Synthesis Kit (Cat # K1612, ThermoScientific). In quantitative polymerase chain reaction (qPCR), glyceraldehyde-3-phosphate dehydrogenase (GAPDH) was used as a control (housekeeping) gene. Master-mix for primers was prepared using 5 μM of combined forward and reverse primer, 2.2 μL of nuclease free water and 5 μL of SYBR Green (Cat # 600883, Agilent Technologies, United States). Next, 8 μL of master-mix and 2 μL of cDNA were mixed in a well in 96-well plate of qPCR (Bio-Rad, United Kingdom). qPCR Plate was processed to CFX96 Touch System Real-Time PCR Detection System (Bio-Rad, United states). Gene expressions were detected using the program: 95 °C for 10 min, 95 °C for 15 s, 60 °C for 30 s and 72 °C for 30 s x (40 cycles), followed by 55 °C for 5 s and 95 °C for 5 s (melting curve). qPCR data was analyzed using delta delta Ct (∆∆Ct) formula to determine the gene expression (mRNA level). Primers for GAPDH, RalA and RalB were designed using Primer3web version 4.1.0. Primers used for different genes in qPCR experiment are listed in Supplementary table 3.

### Assays of MM cell treatment with EVs

#### Assay of MM cell induction toward nephrogenesis by EVs

To determine the functional role of EVs during nephrogenesis, we studied kidney organoids (MM cells) in vitro. In our assay, UB cell line derived EVs (5 μg protein) were added to the 50,000 MM cells to check the effect of EVs on MM cells toward the induction of nephrogenesis. In the assay, MM cells were treated with 6-bromoindirubin-3'-oxime (BIO) (3.5 ng/ml) (Cat # B1686, Sigma Aldrich, United States), PBS, GF (Bmp7 and Fgf2), UB cell line EVs (5 μg protein), UB cell line EVs (5 μg protein) with GF (Bmp7 and Fgf2), UB cell line Sup (5 μg protein) and UB cell line Sup (5 μg protein) with GF (Bmp7 and Fgf2). BIO acts as a nephrogenesis inducer in MM cells and was used as a positive control. Treated MM cells in LoBind Eppendorf tube were centrifuged at 1400 × g for 20 min. LoBind Eppendorf tubes with MM cell pellets (organoids) were incubated overnight at 37 °C. Next day, MM cell organoids were transferred into Trowel type organ culture on 0.1 μm filter in DMEM high glucose media supplemented with 10% FBS and 1% penicillin/streptomycin. The medium was replaced to fresh medium every second day. Bright-field imaging of organoids was done at day 3. Later, MM cell organoids were fixed with 4% PFA for 30 min at day 5 and subjected to immunofluorescence to confirm the presence of nephrons in MM cell organoids.

### Assay of MM cells toward nephrogenesis-competency by EVs

Next, we tested UB cell line EVs for their role in the competency of MM cells for nephrogenesis in the presence of BIO. In this assay, 50,000 MM cells were monolayer-cultured in 48 well plates with UB cell line EVs and other conditions for 16 and 32 h. Conditions included PBS, GF (Bmp7 and Fgf2), UB cell line EVs (5 μg protein), UB cell line EVs (5 μg protein) with GF (Bmp7 and Fgf2) and UB cell line Sup (5 μg protein). One set of conditions was provided with BIO after 16 h of monolayer-culturing, whereas the other set of conditions was provided with BIO after 32 h of monolayer-culturing. In one set after 16 h of monolayer-culturing, the cells were trypsinized, pelleted (1400 × g for 20 min) in LoBind Eppendorf tube, treated with BIO (3.5 ng/ml) and grown overnight with BIO at 37 °C. Next day, pellets (organoids) were transferred into Trowel type organ culture. Organoids were brightfield imaged with Olympus camera after 24 h of culturing in Trowel type organ culture. In the other set after 32 h of monolayer-culturing, the cells were trypsinized and subjected to the same protocol applied to the first set of conditions after 16 h of monolayer-culturing.

### Viability assay for MM cells treated with EVs

To investigate whether UB cell line EVs affect the viability of MM cells or not, MM cells were treated under different conditions for 24 and 48 h according to the protocol described before. Treated MM cells were pelleted at 1400 × g for 20 min followed by incubation overnight at 37 °C. After 24 and 48 h, MM cell pellets were disintegrated by gentle pipetting. Live cells were counted using trypan blue (Cat # T 8154, Sigma, United States) with TC20 Automated Cell Counter.

### Generation of recombinant lentiviruses and transduction of UB cell line

HEK293T cell line was used for the generation of lentiviruses. Lentiviruses were used to KD the expression of RalA, RalB and RalControl gene by shRNA as described previously [25, 36]. Moreover, palmitoylation signal tagged GFP (PalmGFP) vector was used for the generation of lentiviruses. Plasmids for PalmGFP were gifted by Lai´s lab, Academia Sinica, Taiwan and Breakefield´s lab, Department of Neurology and Radiology, Harvard Medical School, United States [21]. In this assay, two mixtures were prepared: a plasmid mixture and a lipofectamine mixture. Plasmid mixture was prepared by using 3 μg DNA in OptiMEM medium (125 μl) containing pVSVG-envelop (Cat # 138479, Addgene) (0.75 μg), pPAX2-Gag/Pol/Rev/Tat (Cat # 12260, Addgene) (0.75 μg) and lentivirus transfer vector (1.5 μg) per transfection. Lipofectamine mixture was prepared by adding 12 ul of Lipofectamine 2000 transfection reagent (Cat # 11668019, ThermoFisher Scientific, United States) to 125 μl of OptiMEM medium per transfection. Plasmid and lipofectamine mixtures were mixed at room temperature. Medium from the confluent HEK 293T cell line was aspirated and fresh prewarmed 1 ml low glucose DMEM medium (Cat # 11885084, ThermoFisher Scientific, United States) containing 0.1% penicillin/streptomycin and 10% FBS was added to HEK293T cell line. Mixture of plasmids and lipofectamine was added to HEK293T cell line. Medium containing the viral particles was collected for the virus mediated transduction of UB cell line. lentiviruses were mixed with Polybrene (8 μg/ml) (Cat # H9268, Sigma Aldrich) in low glucose DMEM medium. Lentiviral particles in the medium were applied to the UB cell line and medium was removed after 48 h. Puromycin (4 μg/ml) (Cat # P9620, Sigma Aldrich) based selection was performed, and after 2 days, living cells were passaged and cultured in Puromycin (1 μg/ml). UB cell lines with the KD of RalA, RalB and RalControl were termed as UB RalA KD cell line, UB RalB KD cell line and UB RalCtl KD cell line, and UB cell line genetically tagged with PalmGFP was termed as UB PalmGFP cell line.

### Fluorescence activated cell sorting

UB PalmGFP cell line cells for GFP expression were sorted by fluorescence activated cell sorting (FACS) using BD FACSAria (BD Biosciences). Sorted cells were collected in DMEM/F-12 medium containing 10% FBS and 1% penicillin/streptomycin. The cells were incubated in 5% CO_2_ at 37 °C.

### Statistical analysis

Mainly, statistical analysis of data was performed by one-way analysis of variance (ANOVA) with multiple comparison test. Levels of significance were described as: *P* > 0.05 (ns), *P* ≤ 0.05 (*), *P* ≤ 0.01 (**) and *P* ≤ 0.001 (***). In viability assay for MM cells treated with EVs, the statistical difference of data sets was analyzed using the Student´s two-tailed t test. Levels of significance were described as: *P* > 0.05 (ns), *P* ≤ 0.05 (*), *P* ≤ 0.01 (**) and *P* ≤ 0.001 (***).

### Supplementary Information

**Additional file 1. **

## Data Availability

The data sets generated and/or analyzed during the current study are available from the corresponding author on request.
