# Supplementary material for: Generation of novel in vitro flexible kidney organoid model to investigate the role of extracellular vesicles in induction of nephrogenesis
Source: Cell Commun Signal. 2023 Dec 18;21:358. doi: 10.1186/s12964-023-01374-z (PMC10726558; doi:10.1186/s12964-023-01374-z)
Supplement: Supplementary file 2 — Additional file 1. [file 12964_2023_1374_MOESM1_ESM.docx]

**Generation of novel in vitro flexible kidney organoid model to investigate the role of extracellular vesicles in induction of nephrogenesis**

Naveed Ahmad^1^, Anatoliy Samoylenko^1^, Ichrak Abene^1^, Eslam Abdelrady^1^, Artem Zhyvolozhnyi^1^, Olha Makieieva^1^, Geneviève Bart^1^, Ilya Skovorodkin^1^ and Seppo J Vainio^1,2,3,4*^

1 Laboratory of Developmental Biology, Faculty of Biochemistry and Molecular Medicine, University of Oulu, 90220 Oulu, Finland

2 Infotech Oulu, University of Oulu, 90014 Oulu, Finland

3 Flagship GeneCellNano, University of Oulu, 90220 Oulu, Finland

4 Kvantum Institute, University of Oulu, 90014 Oulu, Finland

*Corresponding author E-mail:

naveed.ahmad@oulu.fi and Seppo.vainio@oulu.fi

**Supplementary information**


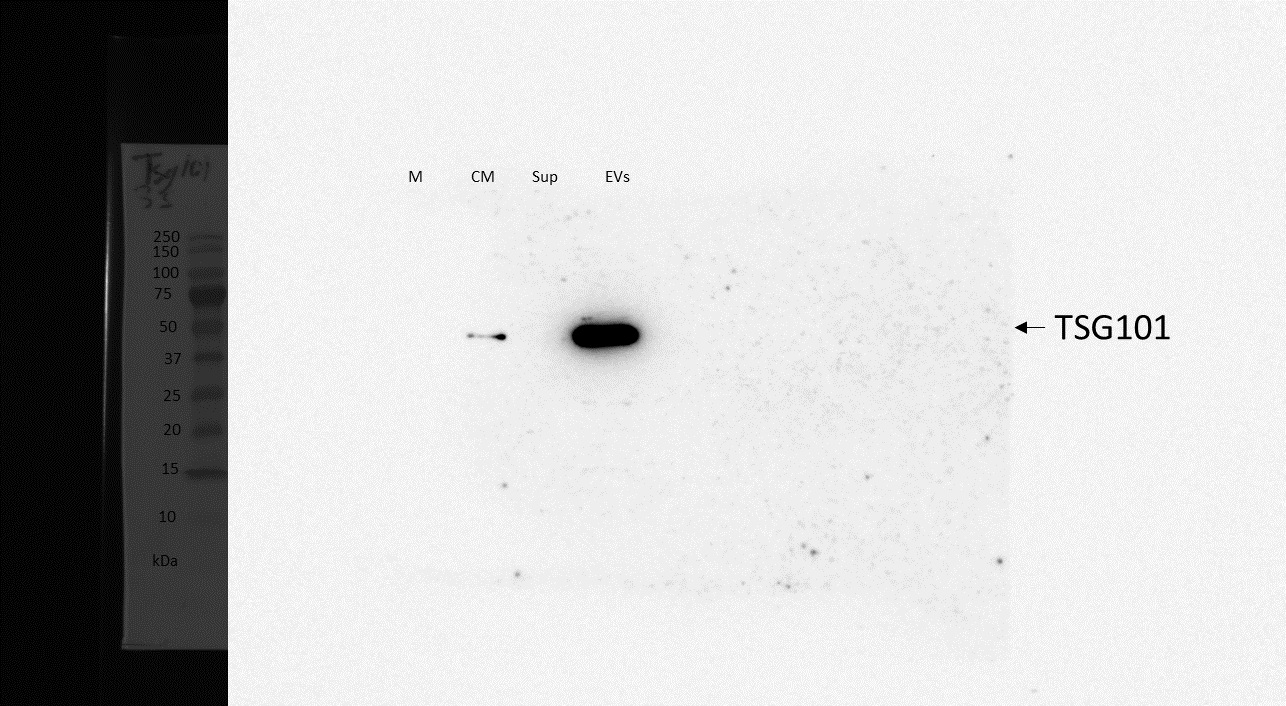
Supplementary Figures

**Supplementary Figure 1. Western blot with anti TSG101 (45 kDa antibody against the standard EV markers in conditioned medium (CM), Sup and EVs.**


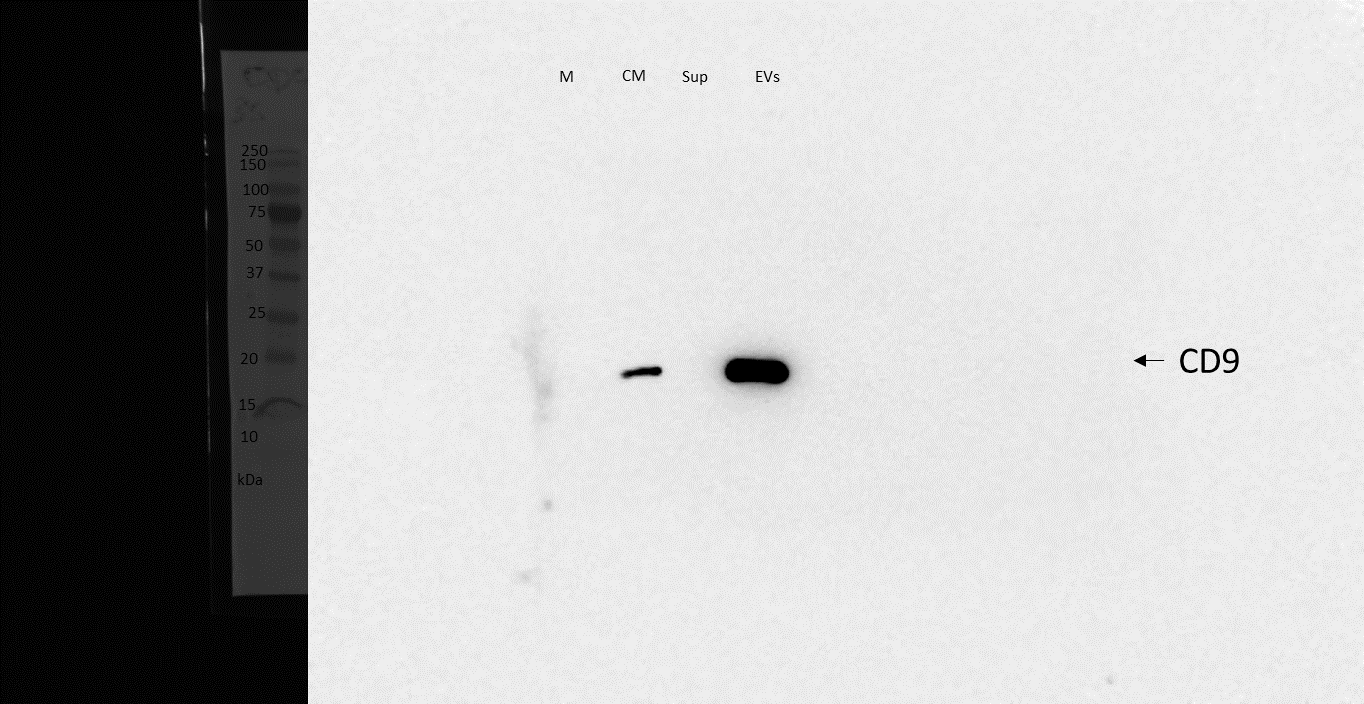


**Supplementary Figure 2. Western blot with anti CD9 (25 kDa) antibody against the standard EV markers in conditioned medium (CM), Sup and EVs.**


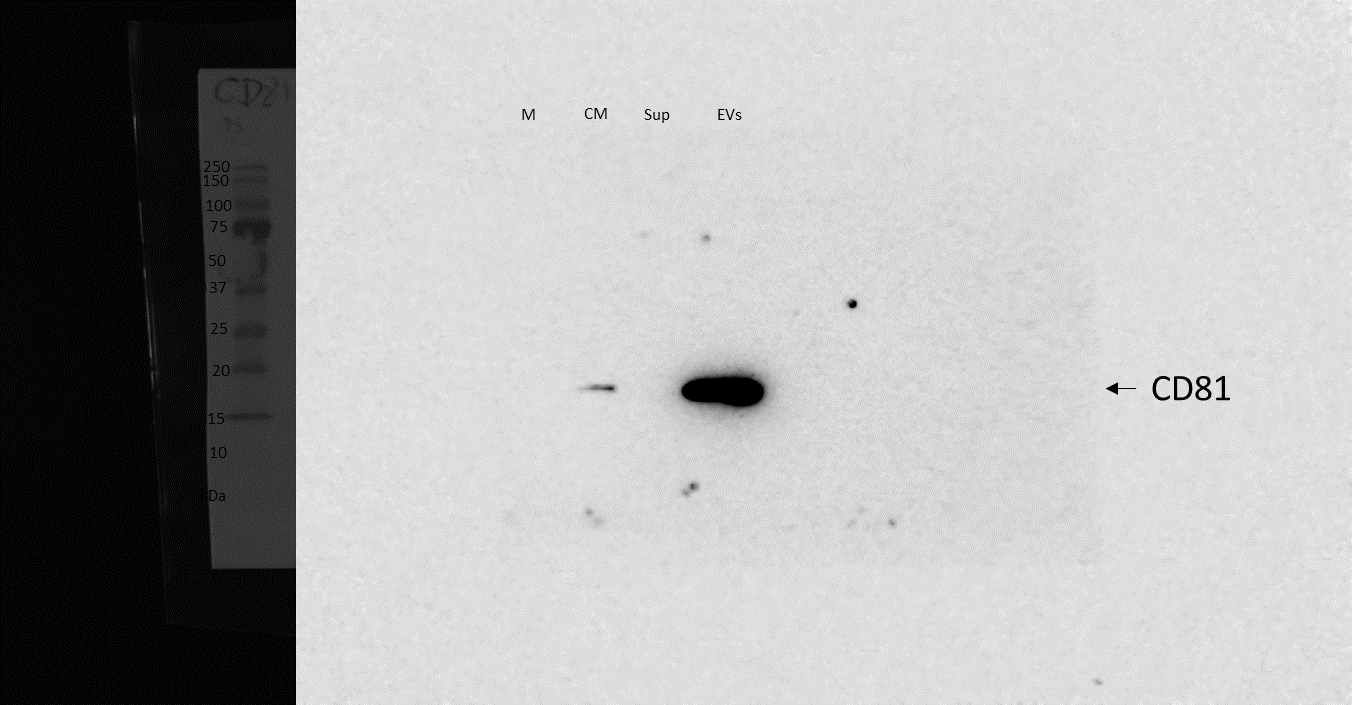


**Supplementary Figure 3. Western blot with anti CD81 (22-26 kDa) antibody against the standard EV markers in conditioned medium (CM), Sup and EVs.**


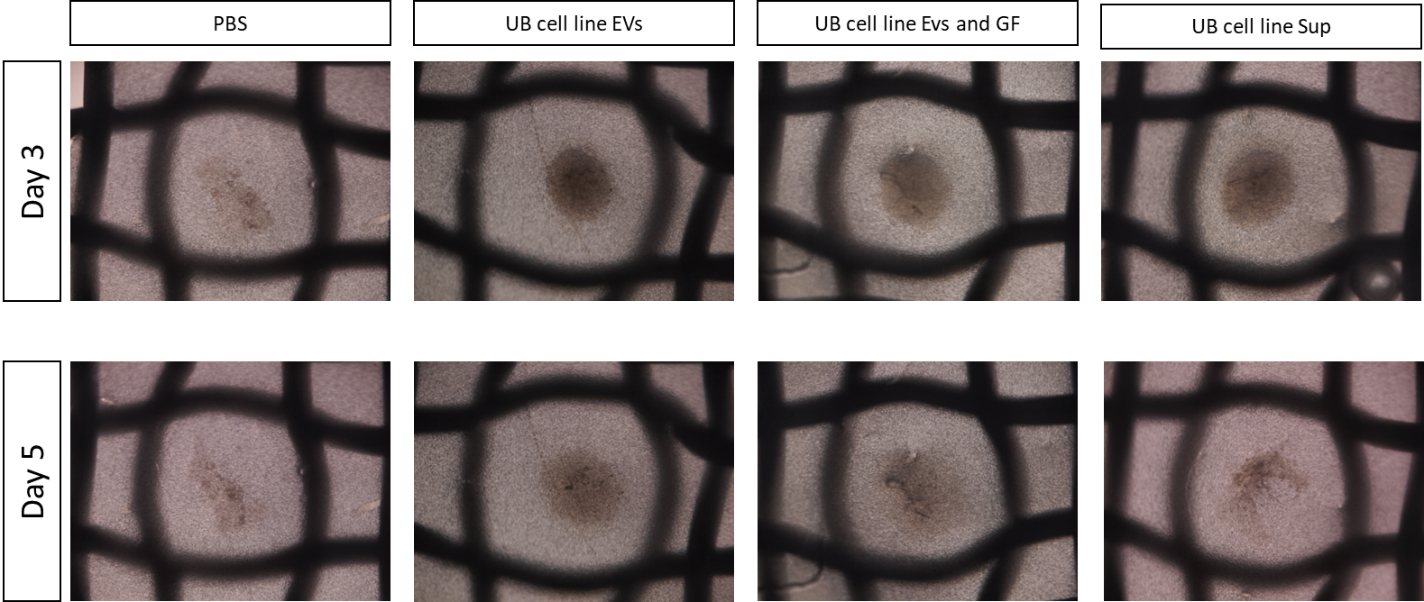
**Supplementary Figure 4. Influence of EVs (2 µg) on MM cells during nephrogenesis. Bright field images of MM cell organoids with different treatments were captured at day 3 and 5.**

**Supplementary tables**

##### One-Way ANOVA with multiple comparison test for the viability (%) of treated MM cells cultured in different conditions for 24 hrs. Level of significance was shown as: P > 0.05 (ns), P ≤ 0.05 (*), P ≤ 0.01 (**) and P ≤ 0.001 (***).

| **Tukey's multiple comparisons test** | **Summary** | **Adjusted P Value** |
| --- | --- | --- |
| BIO vs. PBS | *** | <0,0001 |
| BIO vs. GF | *** | <0,0001 |
| BIO vs. UB cell line EVs | *** | <0,0001 |
| BIO vs. UB cell line EVs and GF | ** | 0,0037 |
| BIO vs. UB cell line Sup | *** | <0,0001 |
| BIO vs. UB cell line Sup and GF | *** | <0,0001 |
| PBS vs. GF | * | 0,0132 |
| PBS vs. UB cell line EVs | ns | 0,1592 |
| PBS vs. UB cell line EVs and GF | *** | 0,0001 |
| PBS vs. UB cell line Sup | ns | 0,8687 |
| PBS vs. UB cell line Sup and GF | ** | 0,0082 |
| GF vs. UB cell line EVs | ns | 0,787 |
| GF vs. UB cell line EVs and GF | ns | 0,1832 |
| GF vs. UB cell line Sup | ns | 0,1191 |
| GF vs. UB cell line Sup and GF | ns | >0,9999 |
| UB cell line EVs vs. UB cell line EVs and GF | * | 0,0155 |
| UB cell line EVs vs. UB cell line Sup | ns | 0,7403 |
| UB cell line EVs vs. UB cell line Sup and GF | ns | 0,6399 |
| UB cell line EVs and GF vs. UB cell line Sup | ** | 0,0011 |
| UB cell line EVs and GF vs. UB cell line Sup and GF | ns | 0,2737 |
| UB cell line Sup vs. UB cell line Sup and GF | ns | 0,0758 |

##### One-Way ANOVA with multiple comparison test for the viability (%) of treated MM cells cultured in different conditions for 48 hrs. Level of significance was shown as: P > 0.05 (ns), P ≤ 0.05 (*), P ≤ 0.01 (**) and P ≤ 0.001 (***).

| **Tukey's multiple comparisons test** | **Summary** | **Adjusted P Value** |
| --- | --- | --- |
| BIO vs. PBS | *** | <0,0001 |
| BIO vs. GF | *** | 0,0002 |
| BIO vs. UB cell line EVs | *** | <0,0001 |
| BIO vs. UB cell line EVs and GF | * | 0,0143 |
| BIO vs. UB cell line Sup | *** | <0,0001 |
| BIO vs. UB cell line Sup and GF | *** | 0,0006 |
| PBS vs. GF | *** | 0,0006 |
| PBS vs. UB cell line EVs | ns | 0,0738 |
| PBS vs. UB cell line EVs and GF | *** | <0,0001 |
| PBS vs. UB cell line Sup | ns | 0,8609 |
| PBS vs. UB cell line Sup and GF | *** | 0,0002 |
| GF vs. UB cell line EVs | ns | 0,159 |
| GF vs. UB cell line EVs and GF | ns | 0,2691 |
| GF vs. UB cell line Sup | ** | 0,0051 |
| GF vs. UB cell line Sup and GF | ns | 0,9968 |
| UB cell line EVs vs. UB cell line EVs and GF | ** | 0,0022 |
| UB cell line EVs vs. UB cell line Sup | ns | 0,4888 |
| UB cell line EVs vs. UB cell line Sup and GF | ns | 0,0604 |
| UB cell line EVs and GF vs. UB cell line Sup | **** | <0,0001 |
| UB cell line EVs and GF vs. UB cell line Sup and GF | ns | 0,5538 |
| UB cell line Sup vs. UB cell line Sup and GF | ** | 0,0018 |

##### Sequences of the primers (5’ – 3’) used for qPCR experiments to measure mRNA levels.

**Gene Forward primer Reverse primer**

*Gapdh* AGAACATCATCCCTGCATCC CAGTGAGCTTCCCGTTCAG

*RalA* TCTTAGATACAGCGGGGCAG CTGCGAAGGACTCCATCTCT

*RalB* CACGAGTCTTTCACAGCCAC ATGTCTCCACGTACTGCACA

##### Proteins in EVs involved in different signaling pathways related to nephrogenesis

| **Wnt signaling pathway**  **UniProt ID Protein** | |
| --- | --- |
| Q9WV60 | Glycogen synthase kinase-3 beta |
| Q99JB2 | Stomatin-like protein 2, mitochondrial |
| Q9WTR5 | Cadherin-13 |
| O54833 | Casein kinase II subunit alpha' |
| P62874 | Guanine nucleotide-binding protein G(I)/G(S)/G(T) subunit beta-1 |
| P21278 | Guanine nucleotide-binding protein subunit alpha-11 |
| P62880 | Guanine nucleotide-binding protein G(I)/G(S)/G(T) subunit beta-2 |
| Q9DAS9 | Guanine nucleotide-binding protein G(I)/G(S)/G(O) subunit gamma-12 |
| Q61151 | Serine/threonine-protein phosphatase 2A 56 kDa regulatory subunit epsilon isoform |
| Q80SZ7 | Guanine nucleotide-binding protein G(I)/G(S)/G(O) subunit gamma-5 |
| P21279 | Guanine nucleotide-binding protein G(q) subunit alpha |
| P67871 | Casein kinase II subunit beta |
| Q9JMK2 | Casein kinase II subunit beta |
| **VEGF signaling pathway**  **UniProt ID Protein** | |
| P47811 | Mitogen-activated protein kinase 14 |
| Q63844 | Mitogen-activated protein kinase 3 |
| P08556 | GTPase NRas |
| P47713 | Cytosolic phospholipase A2 |
| P34152 | Focal adhesion kinase 1 |
| **TGF-beta signaling pathway**  **UniProt ID Protein** | |
| Q62432 | Mothers against decapentaplegic homolog 2 |
| P47811 | Mitogen-activated protein kinase 14 |
| P45878 | Peptidyl-prolyl cis-trans isomerase FKBP2 |
| P10833 | Ras-related protein R-Ras |
| Q63844 | Mitogen-activated protein kinase 3 |
| P08556 | GTPase NRas |
| P62071 | Ras-related protein R-Ras2 |
| P61027 | Ras-related protein Rab-10 |
| Q9CUN6 | E3 ubiquitin-protein ligase SMURF1 |
| **PDGF signaling pathway**  **UniProt ID Protein** | |
| Q9WV60 | Glycogen synthase kinase-3 beta |
| Q63844 | Mitogen-activated protein kinase 3 |
| P08556 | GTPase NRas |
| P46638 | Ras-related protein Rab-11B |
| Q7TT37 | Elongator complex protein 1 |
| Q91Z67 | SLIT-ROBO Rho GTPase-activating protein 2 |
| **Notch signaling pathway**  **UniProt ID Protein** | |
| O35598 | Disintegrin and metalloproteinase domain-containing protein 10 |
| P49769 | Presenilin-1 |
| P57716 | Nicastrin |
| **Integrin signaling pathway**  **UniProt ID Protein** | |
| Q8BFZ3 | Beta-actin-like protein 2 |
| P05480 | Neuronal proto-oncogene tyrosine-protein kinase Src |
| Q8BU31 | Ras-related protein Rap-2c |
| P61226 | Ras-related protein Rap-2b |
| Q9D898 | Actin-related protein 2/3 complex subunit 5-like protein |
| Q62470 | Integrin alpha-3 |
| P62835 | Ras-related protein Rap-1A |
| P11688 | Integrin alpha-5 |
| P70460 | Vasodilator-stimulated phosphoprotein |
| Q99JI6 | Ras-related protein Rap-1b |
| P62331 | ADP-ribosylation factor 6 |
| P10833 | Ras-related protein R-Ras |
| Q63844 | Mitogen-activated protein kinase 3 |
| P43406 | Integrin alpha-V |
| O55222 | Integrin-linked protein kinase |
| P08556 | GTPase NRas |
| P61211 | ADP-ribosylation factor-like protein 1 |
| Q9Z0T9 | Integrin beta-6 |
| Q62159 | Rho-related GTP-binding protein RhoC |
| P34152 | Focal adhesion kinase 1 |
| Q9ES46 | Beta-parvin |
| Q80ZJ1 | Ras-related protein Rap-2a |
| Q62469 | Integrin alpha-2 |
| Q9CPW4 | Actin-related protein 2/3 complex subunit 5 |
| **Hh signaling pathway**  **UniProt ID Protein** | |
| Q9WV60 | Glycogen synthase kinase-3 beta |
| P12367 | cAMP-dependent protein kinase type II-alpha regulatory subunit |
| Q9JMK2 | Casein kinase I isoform epsilon |
| **FGF signaling pathway**  **UniProt ID Protein** | |
| P47811 | Mitogen-activated protein kinase 14 |
| Q63844 | Mitogen-activated protein kinase 3 |
| P08556 | GTPase NRas |
| Q61151 | Serine/threonine-protein phosphatase 2A 56 kDa regulatory subunit epsilon isoform |
| **EGF receptor signaling pathway**  **UniProt ID Protein** | |
| P47811 | Mitogen-activated protein kinase 14 |
| P10833 | Ras-related protein R-Ras |
| Q63844 | Mitogen-activated protein kinase 3 |
| P08556 | GTPase NRas |
| Q61151 | Serine/threonine-protein phosphatase 2A 56 kDa regulatory subunit epsilon isoform |
| O08759 | Ubiquitin-protein ligase E3A |
| P62071 | Ras-related protein R-Ras2 |
| P84096 | Rho-related GTP-binding protein RhoG |
| Q04690 | Neurofibromin |
| **Cytoskeletal regulation by Rho GTPase**  **UniProt ID Protein** | |
| Q8BFZ3 | Beta-actin-like protein 2 |
| Q9D6F9 | Tubulin beta-4A chain |
| P70460 | Vasodilator-stimulated phosphoprotein |
| Q7TMM9 | Tubulin beta-2A chain |
| Q61879 | Myosin-10 |
| Q9CWF2 | Tubulin beta-2B chain |
| Q62159 | Rho-related GTP-binding protein RhoC |
| Q9CPW4 | Actin-related protein 2/3 complex subunit 5 |
| **Cadherin signaling pathway**  **UniProt ID Protein** | |
| Q8BFZ3 | Beta-actin-like protein 2 |
| P05480 | Neuronal proto-oncogene tyrosine-protein kinase Src |
| Q9WV60 | Glycogen synthase kinase-3 beta |
| Q04736 | Tyrosine-protein kinase Yes |
| Q9WTR5 | Cadherin-13 |
| P35821 | Tyrosine-protein phosphatase non-receptor type 1 |
| O54833 | Casein kinase II subunit alpha' |
| P67871 | Casein kinase II subunit beta |

##### Proteins in supernatant involved in different nephrogenesis related signaling pathways

| **Wnt signaling pathway**  **UniProt ID Protein** | |
| --- | --- |
| Q80TF3 | Protocadherin-19 |
| O88338 | Cadherin-16 |
| O88572 | Low-density lipoprotein receptor-related protein 6 |
| **TGF-beta signaling pathway**  **UniProt ID Protein** | |
| Q04998 | Inhibin beta A chain |
| **PDGF signaling pathway**  **UniProt ID Protein** | |
| P42227 | Signal transducer and activator of transcription 3 |
| **Notch signaling pathway**  **UniProt ID Protein** | |
| Q01705 | Neurogenic locus notch homolog protein 1 |
| **Integrin signaling pathway**  **UniProt ID Protein** | |
| Q60847 | Collagen alpha-1(XII) chain |
| **Integrin signaling pathway**  **UniProt ID Protein** | |
| Q60847 | Collagen alpha-1(XII) chain |
| **FGF signaling pathway**  **UniProt ID Protein** | |
| Q7TNP2 | Serine/threonine-protein phosphatase 2A 65 kDa subunit A beta isoform |
| **EGF receptor signaling pathway**  **UniProt ID Protein** | |
| P42227 | Signal transducer and activator of transcription 3 |
| **Cytoskeletal regulation by Rho GTPase**  **UniProt ID Protein** | |
| O88643 | Serine/threonine-protein kinase PAK 1 |
| **Cadherin signaling pathway**  **UniProt ID Protein** | |
| Q80TF3 | Protocadherin-19 |
| O88338 | Cadherin-16 |
